# Proteomic and Transcriptomic Analyses of Swine Pathogen *Erysipelothrix rhusiopathiae* Reveal Virulence Repertoire

**DOI:** 10.1371/journal.pone.0159462

**Published:** 2016-08-01

**Authors:** Yufeng Li, Yao Zou, Yuting Xia, Juan Bai, Xianwei Wang, Ping Jiang

**Affiliations:** Key Laboratory of Bacteriology, Ministry of Agriculture, College of Veterinary Medicine, Nanjing Agricultural University, Nanjing, 210095, China; University of Osnabrueck, GERMANY

## Abstract

*E*. *rhusiopathiae* is the causative agent of erysipelas in animals and erysipeloid in humans, but its pathogenicity is poorly understood. To identify virulence factors associated with *E*. *rhusiopathiae* and screen engineered vaccine candidates, we used proteomics and transcriptomics to compare the highly virulent strain HX130709 with an isogenic avirulent derivative, HX130709a. 1,299 proteins and 1,673 transcribed genes were identified and 1,292 of the proteins could be associated with genes. In a comparison between HX130907 and HX130709a, 168 proteins and 475 genes exhibited differences in regulation level. Among these, levels for 61 proteins and transcripts were positively or negatively correlated. Gene Ontology (GO) analysis suggests that many of the down-regulated proteins in the attenuated strain have catalytic or binding functions. Potential protein-protein interactions suggest that some of the down-regulated proteins may regulate PTS, GMP synthase and ribosomal proteins. Morphological results showed that HX130709 and HX130709a have similar colony and capsule morphology. Growth curves and pyruvate measurements suggest that TCA cycle and saccharide phosphorylation levels were decreased and gluconeogenesis was increased in HX130709a. Our study confirms that SpaA and neuraminidase, but not hyaluronidase and capsule, are associated with virulence in *E*. *rhusiopathiae*. We conclude that the virulence of *E*. *rhusiopathiae* may be associated with slow reactions of the TCA cycle and down-regulation of selected proteins.

## Introduction

*Erysipelothrix rhusiopathiae* (*E*. *rhusiopathiae*) is a small gram-positive, slender, and straight rod-shaped bacterium that can cause erysipelas in swine and other animals, including sheep, fish, reptiles and birds. *E*. *rhusiopathiae* is also an important pathogen in humans and is the causative agent of erysipeloid, a skin disease [1]. The bacterium can be isolated from sick and healthy animals (pork, seafood, chicken) and from environment in which they live [2, 3]. *E*. *rhusiopathiae* belong to the genus *Erysipelothrix* along with *E*. *tonsillarum* and two other unnamed species [4]. Among the 15 serotypes of *E*. *rhusiopathiae*, serotypes 1a and 2 have the greatest impact on the swine industry [1, 5–9]. Swine erysipelas has caused serious losses in the swine industries of North America, Europe, Asia, and Australia. Swine erysipelas occurs in three forms: acute, subacute, and chronic. The characteristics of acute erysipelas in swine are sudden death or general signs of septicemia. Sub-acute erysipelas causes urticarial or diamond-skin lesions that appear as early as the second or third day after infection. The chronic form of infection can develop from acute or subacute disease, and can be characterized by localized arthritis or proliferative pathological changes in the heart (endocarditis) [10].

*E*. *rhusiopathiae* infection in humans is mostly occupationally related, those most prone include butchers, abattoir workers, veterinarians, farmers, fishermen, fish-handlers and housewives [11]. Clinical manifestations in humans are highly similar to those in swine and occur in a localized cutaneous form (erysipeloid), a generalized cutaneous form, and a septicemic form associated with endocarditis [10].

The virulence of *E*. *rhusiopathiae* varies considerably among different serotypes. Though the mechanisms of pathogenicity are poorly understood, and no toxin has been identified in this organism, several candidate virulence factors have been identified, including neuraminidase [12], capsular antigens [13], RspA and RspB[14], and the 64-66kDa antigen[15]. However, because these studies compared highly virulent with moderate or low virulence strains, the complete virulence repertoire may not have been revealed. In the present study, a highly virulent strain (HX130709) was attenuated by 70 passages on agar medium containing a gradually increasing concentration (0.0025% to 0.03%) of acriflavine dye, with attenuation confirmed by mouse pathogenicity. Proteomic and transcriptomic analyses were then used to identify differences between the attenuated strain HX130709a and its parent, HX130709, to reveal the virulence repertoire of *E*. *rhusiopathiae*.

## Materials and Methods

### Reagents

Reagents were obtained from the following sources: 2-D Quant Kit (GE Healthcare), TEAB (Applied Biosystems, Milan Italy), Trypsin Gold (Promega, Madison WI, USA), iTRAQ Reagents -8plex Chemistry (Applied Biosystems), TruSeq SBS KIT-HS, TruSeq PE Cluster Kit and TruSeq SR Cluster Kit (Illumina), TRIzol (Invitrogen), CytoTox 96 Non-Radioactive Cytotoxicity Assay (Promega), mouse GAPDH antibody (GeneTex), DNase I (Ambion), Terminator 5΄-phosphate-dependent exonuclease (Epicentre).

### Bacterial strains and culture conditions

HX130709, a highly virulent strain isolated from a case of septicemia, has been confirmed by PCR and is pathogenic in mice. To generate HX130709a, HX130709 was attenuated by 70 passages on BHI-T80 agar containing an acriflavine dye (0.0025% to 0.03%). The reduced pathogenicity of the attenuated strain was confirmed in mice[16]. Animal experiments were approved by the Institutional Animal Care and Ethics Committee of Nanjing Agricultural University (Approval No. IACECNAU20100902).

### Sample preparation

Brain Heart infusion agar and broth supplemented with 0.1% Tween 80 were used for bacterial cultivation. Pure cultures of strains HX130709 and HX130709a were grown in 500 ml of BHI broth for 4 h and 10 h, respectively. Cultures were harvested by centrifugation at 4000 rpm for 15 min at 4°C and washed three times in 1 × PBS buffer. Washed cells were collected in sterile tubes, flash frozen in liquid nitrogen, and submitted to BGI tech for proteomic and transcriptomic analyses. Three biological replicates were prepared independently for each sample.

### Quantitative transcriptomics (RNA-seq)

#### RNA isolation and mRNA purification

Total RNA was isolated with TRIzol reagent using the standard protocol, and dissolved in 200 μL RNase-free water. The concentration of total RNA was determined using a NanoDrop spectrophotometer (Thermo Scientific, USA), and the RNA integrity value (RIN) was determined using the RNA 6000 Pico LabChip and an Agilent 2100 Bioanalyzer (Agilent, USA). Total RNA was incubated with 10 U DNase I at 37°C for 1 h, and then nuclease-free water was added to bring the sample volume to 250 μL. Messenger RNA was further purified by digesting ribosomal RNA and tRNA with Terminator 5΄-phosphate-dependent exonuclease. The resulting RNA samples were quantified using a DU800 spectrophotometer (Beckman Coulter, USA) and mixed with fragmentation buffer to generate short mRNA fragments.

#### cDNA synthesis and Illumina sequencing

cDNA was synthesized using the mRNA fragments as templates. The short cDNA fragments were purified and resuspended in EB buffer for end repair and single nucleotide A (adenine) addition, then ligated to adapters. After agarose gel electrophoresis, appropriately sized products were selected as templates for PCR amplification. During the QC steps, the Agilent 2100 Bioanalyzer and ABI StepOnePlus Real-Time PCR System were used to monitor sample library quantity and quality. The library was sequenced using the Illumina HiSeq^™^ 2000 high-throughput sequencing system.

#### Bioinformatics analysis

Sequencing reads were mapped to the reference genome Fujisawa (NC_015601) using BLASTN with a threshold *e* value of 0.00001 and the “-F F” parameter [17], which allowed alignments with up to two mismatches. Reads that mapped to rRNA genes or that failed to align using these parameters were excluded from further analysis. The read totals were expressed as RPKM (Reads Per Kilo bases per Million reads) [18] for each gene, and then differently regulated genes were identified using the DEGseq package and the MARS (MA-plot-based method with Random Sampling model) method [19]. We used FDR ≤ 0.001 and an absolute value of log_2_Ratio ≥ 1 as thresholds to judge the significance of differences in gene expression.

### Quantitative proteomics

#### Protein preparation

Harvested bacteria were washed three times with ice-cold phosphate-buffered saline (137 mM NaCl, 2.7 mM KCl, 10.1 mM Na_2_HPO_4_, 1.8mM KH_2_PO_4_, pH 7.4). The supernatant was discarded after the final centrifugation at 12,000 × g for 30 min. The pellets were extracted with lysis buffer (7 M Urea, 2 M Thiourea, 4% CHAPS, 40 mM Tris-HCl, pH 8.5) with a final concentration of 1mM PMSF and 2mM EDTA. After 5 min of vortexing, DTT was added to the samples to a final concentration of 10 mM. The samples were sonicated at 200 W for 15 min and then centrifuged at 4°C, 30,000 × g for 15 min. The samples were mixed well with 5× volume of ice-cold acetone containing 10% (v/v) TCA and incubated at –20°C overnight. After centrifugation at 4°C, 30,000 × g, the supernatant was discarded. The precipitate was washed with ice-cold acetone three times. The pellet was vacuum-dried and dissolved in lysis buffer (7M urea, 2 M thiourea, 4% NP40, 20mM Tris-HCl, pH 8.0–8.5). The samples were sonicated at 200 W for 15 min and centrifuged at 4°C, 30,000 × g for 15 min, and then the supernatant was transferred to a new tube. To reduce disulfide bonds in proteins, 10 mM DTT (final concentration) was added and samples were incubated at 56°C for 1 h. Subsequently, 55 mM IAM (final concentration) was added to block the cysteines, and samples were incubated for 1 h in the dark. The supernatant was mixed well with 5× volume of ice-cold acetone for 2 h at –20°C to precipitate proteins. After centrifugation at 4°C, 30,000 × g, the supernatant was discarded and the pellet was vacuum-dried for 5 min. The samples were then dissolved in 500 μL 0.5 M TEAB and sonicated at 200 W for 15 min. Finally, samples were pelleted at 4°C, 30,000 × g for 15 min. The supernatant was transferred to a new tube and protein content quantified. Protein preparations were stored at –80°C for later analysis.

#### iTRAQ (Isobaric tag for relative and absolute quantitation) labeling and SCX fractionation

100 μg of total protein was withdrawn from each sample and then digested at 37°C for 16 hours with Trypsin Gold, at a protein: trypsin ratio of 30: 1. After digestion, peptides were vacuum-dried and re-dissolved in 0.5M TEAB, then processed with 8-plex iTRAQ reagent, according to the manufacturer’s protocol. Briefly, one unit of iTRAQ reagent was thawed and mixed with 24 μL isopropanol. The peptides labeled with the isobaric tags were pooled then dried by vacuum centrifugation.

SCX chromatography was performed using a LC-20AB HPLC Pump system (Shimadzu, Kyoto, Japan). The peptide mixtures labeled with isobaric tags were reconstituted in 4 mL buffer A (25 mM NaH2PO4 in 25% ACN, pH 2.7) and loaded onto a 4.6×250 mm Ultremex SCX column containing 5-μm particles (Phenomenex). The peptides were eluted with a gradient of buffer A for 10 min, at a flow rate of 1 mL/min, then eluted with 5–60% buffer B (25mM NaH_2_PO4, 1 M KCl in 25% ACN, pH 2.7) for 27 min, and then with 60–100% buffer B for 1 min. Prior to the next sample injection, the system was maintained in 100% buffer B for 1 min, then equilibrated with buffer A for 10 min. Elution was monitored and fractions were pooled every 1 min. The eluted peptides were grouped into 20 fractions and desalted with a Strata X C18 column (Phenomenex) then vacuum-dried.

#### LC-ESI-MS/MS analysis based on Q EXACTIVE

Each fraction was re-dissolved in buffer A containing 2% ACN and 0.1% FA and centrifuged at 20000 × g for 10 min. The final concentration of peptide was about 0.5 μg/μL. 10 μL supernatant was loaded by autosampler onto a 2 cm C18 trap column for analysis in a LC-20AD nanoHPLC (Shimadzu, Kyoto, Japan). The peptides were eluted onto an analytical C18 column (inner diameter 75 μm) packed in-house. The samples were loaded at 8 μL/min for 5 min, then the column was run with a gradient from 2% to 35% in buffer B (98% ACN, 0.1% FA) at 300 nL/min for 35 min. A linear gradient to 80% was run through the column for 2 min, 80% buffer B was maintained for 4 min then returned to 5% for 1 min.

The peptides subjected to nanoelectrospray ionization were analyzed by tandem mass spectrometry (MS/MS) in a Q EXACTIVE instrument (Thermo Fisher Scientific, San Jose, CA) coupled online with the HPLC. Orbitrap was used to detect intact peptides at a resolution of 70,000. MS/MS was used to select peptides in high-energy collision dissociation (HCD) operating mode with a normalized collision energy setting of 27.0 and Orbitrap was set at a resolution of 17,500. The 15 most abundant precursor ions above a threshold ion count of 20,000 in the MS scan with a subsequent Dynamic Exclusion duration of 15 s were analyzed with a data-dependent procedure that alternated between one MS scan followed by 15 MS/MS scans. The electrospray voltage was set as 1.6 kV. Automatic gain control (AGC) was applied to optimize the spectra generated by the Orbitrap. The AGC target for full MS was 3e6 and 1e5 for MS2. For MS scans, the m/z scan range was between 350 and 2,000 Da. For MS2 scans, the m/z scan range was between 100 and 1,800.

#### Data analysis

Raw data files obtained from the Orbitrap were converted into MGF format using Proteome Discoverer 1.2 (PD 1.2, Thermo), [5600 msconverter]. Proteins were identified using the Mascot search engine (Matrix Science, London, UK; version 2.3.02) against a database (downloaded from NCBI http://www.ncbi.nlm.nih.gov/protein/?term=txid1648[Organism:exp]) containing 3,409 sequences. For protein identification, a mass tolerance of 20 Da (ppm) was used for intact peptide masses and 0.05 Da for fragmented ions, and one missing trypsin cleavage was permitted in the trypsin digests. Gln->pyro-Glu (N-term Q), Oxidation (M), and Deamidated (NQ) were specified as potential modifications, and Carbamidomethyl (C), iTRAQ8plex (N-term), and iTRAQ8plex (K) were set as invariable modifications. Peptide charge states were set to +2 and +3. We included an automatic decoy database search as part of the analysis. In this option, whenever a protein sequence from the target database is tested, a random decoy sequence of the same length is also tested. To reduce false positives, proteins required support from at least one unique peptide, and only peptides that exceeded “identity” at 99% confidence were accepted.

For protein quantitation, at least two unique peptides were required. The median ratio by Mascot was used to weight and normalize quantitative protein ratios. Proteins with p-values < 0.05 and fold changes >1.5 were considered to be significantly different between samples.

#### Functional annotation

The functional annotation of proteins was conducted using the Blast2GO application and the non-redundant (NR) protein database at NCBI. The KEGG database (http://www.genome.jp/kegg/) and the COG protein database (http://www.ncbi.nlm.nih.gov/COG/) were used to classify and group the identified proteins.

### Growth curves

A single colony of HX130709 (F0) and HX130709a (F70) was used to inoculate overnight cultures in BHI medium. 300 μL overnight culture was inoculated into 30 mL BHI medium and incubated at 200 rpm. 500 μL samples were collected in triplicate after 1 h and centrifuged at 12,000 rpm for 3 min. The supernatant was discarded, pelleted bacteria were re-suspended in an equal volume of PBS, and optical density (OD600 nm) was measured.

### Morphological observations

HX130709 and the attenuated derivative strain HX130709a were streaked onto BHI agar containing 0.1% Tween 80, incubated overnight, and colony morphology observed. Bacteria cultured to log phase were pelleted and fixed with 2.5% glutaraldehyde and then sectioned. Thin sections were stained with uranyl acetate and lead citrate and examined with a Hitachi H-7500 transmission electron microscope.

### Lactate dehydrogenase activity

Bacteria were cultured to mid-logarithmic phase in BHI medium at 37°C. Triplicate samples were collected for each of 3 time points for the virulent strain HX130709 (5h, 6h, 7h) and the attenuated strain HX13079a (8h, 9h, 10h). Each 10 mL sample was centrifuged at 6,000 rpm for 10 min, then washed in PBS 3 times. Pelleted bacteria were resuspended in 500 μL PBS and lysed by sonication. The lysate was centrifuged at 12,000 rpm for 1 min and supernatant collected. 50 μL supernatant was added to wells in 96-well plates, lactate dehydrogenase (LDH) substrate was added, and the plates were incubated at 37°C for 30 min, following the protocol accompanying the CytoTox 96 Non-Radioactive Cytotoxicity Assay kit. Color development was halted by the addition of stop solution and optical density at 490 nm (OD490) was recorded using an ELISA plate reader (BioTek, USA). In parallel, sampled bacteria were plated at a final dilution of 10^−7^ on BHI agar plates to determine bacterial concentration (colony forming units, CFU). Results were expressed as LDH/cfu.

## Results and Discussion

### Summary of RNA-seq and iTRAQ data

Transcriptome data were obtained by high-throughput RNA-sequencing as described in materials and methods. Three RNA samples were analyzed from HX130709 and its attenuated derivative HX130709a (six samples total). After excluding low-scoring sequence reads, the average read length was 90 bp and the total reads obtained were 3,675,074, 4,692,139 and 3,839,099 for samples HX130709-1, -2, -3 respectively, and 3,251,469, 4,755,689 and 5,151,752 for samples HX130709a-1, -2, -3, respectively. Correlation analysis among the replicates of HX130709, and among the replicates of HX130709a, generated Pearson’s correlation coefficients over 0.95 except in two cases. Because the coefficients for HX130709a-1 vs. HX130709a-2 (0.206) and HX130709a-1 vs. HX130709a-3 (0.2008) were unacceptably low, the HX130709a-1 data set was discarded. In spite of this loss, the mean sequencing coverage for both strains was 193–259 fold. Reads were mapped to the *E*. *rhusiopathiae* genome sequence (Fujisawa) using Soap (v2.01). The results indicated that the transcriptional percentages for ORFs encoded by the Fujisawa strain were 96.37% and 84.58% for HX130709 and HX130709a, respectively. Surprisingly, no SNPs were found in HX130709 or HX130709a. Gene differential regulation was analyzed using the DEGseq software package. Out of 1,673 genes examined, 197 genes exhibited increased transcription in HX130709a and 278 genes exhibited decreased transcription, relative to the levels in HX130709 (S1 Table).

Three biological replicates for each strain were included in the iTRAQ experiment comparing virulent HX130709 and avirulent HX130709a. After trypsinization and labeling with distinct isobaric tags, protein fragments in the six samples were separated and identified by LC-ESI-MS/MS. Because the error distribution analysis among the replicates of HX130709, and among the replicates of HX130709a, showed that the mean errors for HX130709a-1 with replicas -2 and -3 were 1.37 and 1.43, respectively, the data obtained from HX130709a-1 was again set aside. In the five remaining samples, a total of 312,350 mass spectra were generated. After excluding low-scoring spectra, 79,681 unique spectra that matched to specific peptides were obtained. Mascot analysis identified 12,154 peptides, 11,655 unique peptides, and 1,299 proteins in total, in the five samples (S1 File). Covariance distribution analysis for these samples yielded a mean covariance of 0.063 (S1 Fig), indicating good reproducibility between biological replicates in each group. A protein with ≥ 1.5-fold difference and p-value ≤ 0.05 was regarded as being differentially regulated. Relative to protein levels in the virulent strain HX130709, the abundance of 101 proteins increased, and 67 proteins decreased, in HX130709a (Table 1).

**Table 1:** Differentially regulated proteins identified in proteome and transcriptome (HX130709a/HX130709). Protein level differences with p-values < 0.05, and fold changes of >1.5 were considered significant.

Accession	Description	Cov	Unique Peptide	ratio	functions	mRNA expression	Gene ID
**Up-regulation proteins (101)**							
**gi|336065729**	hypothetical protein	33	6	2.005	Unknown		
**gi|322464469**	polysaccharide deacetylase	57.2	23	1.563	Predicted xylanase/chitin deacetylase		
**gi|322462985**	LytTr DNA-binding domain protein	35.9	4	1.502	Response regulator of the LytR/AlgR family		
**gi|336065511**	two-component system response regulator	62.2	13	1.949	Response regulator of the LytR/AlgR family	up	ERH_0269
**gi|322463071**	amino acid permease	2.1	1	1.606	Amino acid transporters	up	ERH_0305
**gi|336066470**	hypothetical protein	74.3	4	1.718	Unknown	down	ERH_1233
**gi|336065272**	ferric uptake regulator family protein	58.3	7	1.56	Fe2+/Zn2+ uptake regulation proteins		
**gi|322463447**	ABC transporter, substrate-binding protein, QAT family	44.1	10	6.58	Periplasmic glycine betaine/choline-binding (lipo)protein of an ABC-type transport system (osmoprotectant binding protein)	up	ERH_1627
**gi|336065597**	glutaredoxin-like protein NrdH	75	5	2.394	Thiol-disulfide isomerase and thioredoxins	down	ERH_0356
**gi|336066593**	peptidase, M23B family	3.2	2	1.565	Membrane proteins related to metalloendopeptidases		
**gi|336066734**	spermidine/putrescine ABC transporter ATP-binding protein	70.3	16	2.416	ABC-type spermidine/putrescine transport systems, ATPase components	up	ERH_1498
**gi|336066419**	hypothetical protein	7.1	2	1.666	Unknown		
**gi|322464148**	hypothetical protein HMPREF0357_10136	17.5	5	1.512	Predicted nucleotidyltransferase		
**gi|336066664**	LPXTG-motif cell wall anchor domain-containing protein	9	13	1.535	Unknown		
**gi|489869940**	signal peptidase	37.9	2	2.437	Unknown	down	ERH_0786
**gi|489872223**	PTS cellobiose transporter subunit IIC	6.8	3	6.19	Phosphotransferase system cellobiose-specific component IIC	up	ERH_0219
**gi|322464339**	biotin/lipoate A/B protein ligase family protein	40.9	7	1.554	Lipoate-protein ligase A	down	ERH_0787
**gi|322464475**	helix-turn-helix protein, YlxM/p13 family	27.8	3	1.646	Uncharacterized protein conserved in bacteria		
**gi|322463067**	response regulator receiver domain protein	32.5	6	1.514	Response regulators consisting of a CheY-like receiver domain and a winged-helix DNA-binding domain		
**gi|489869961**	carbamate kinase	30.9	7	1.809	Carbamate kinase	down	ERH_0797
**gi|336066177**	ABC transporter ATP-binding protein	49.2	8	1.662	ABC-type multidrug transport system, ATPase component	up	ERH_0939
**gi|336066253**	formamidopyrimidine-DNA glycosylase	23.4	4	1.916	Formamidopyrimidine-DNA glycosylase		
**gi|336066573**	copper chaperone	71.4	4	1.805	Unknown		
**gi|336065993**	oxaloacetate decarboxylase subunit alpha	38.5	13	4.116	Pyruvate/oxaloacetate carboxyltransferase		
**gi|336066878**	hypothetical protein	13.6	1	2.808	Unknown		
**gi|336066225**	hypothetical protein	8.5	2	1.665	ABC-type uncharacterized transport system, permease component		
**gi|489870987**	thioredoxin	69.6	5	1.964	Thiol-disulfide isomerase and thioredoxins	down	ERH_1500
**gi|322463512**	hypothetical protein HMPREF0357_10813	52.4	5	1.99	Unknown		
**gi|336065984**	triphosphoribosyl-dephospho-CoA synthase	19.6	4	2.114	Triphosphoribosyl-dephospho-CoA synthetase	up	ERH_0746
**gi|322464227**	CAAX amino terminal protease family protein	6.2	2	1.789	Predicted metal-dependent membrane protease		
**gi|336066870**	hypothetical protein	36.1	4	2.983	Unknown		
**gi|336066016**	hypothetical protein	57.3	7	2.395	Uncharacterized conserved protein		
**gi|322463205**	glycerophosphodiester phosphodiesterase family protein	18.6	10	1.705	Glycerophosphoryl diester phosphodiesterase		
**gi|336066010**	prolyl aminopeptidase	30	7	1.914	Predicted hydrolases or acyltransferases (alpha/beta hydrolase superfamily)	up	ERH_0772
**gi|322463949**	hypothetical protein HMPREF0357_11250	57.6	6	1.981	Predicted DNA-binding protein with PD1-like DNA-binding motif		
**gi|336066178**	hypothetical protein	9.7	5	1.987	Unknown		
**gi|336066448**	TetR family transcriptional regulator	34.8	6	1.53	Transcriptional regulator		
**gi|336065561**	Crp/Fnr family transcriptional regulator	12.1	2	2.196	Predicted transcriptional regulators		
**gi|336065842**	hypothetical protein	40	2	2.474	Unknown	down	ERH_0602
**gi|336065758**	tyrosine recombinase XerC	35.1	8	1.617	Site-specific recombinase XerD		
**gi|336065996**	oxaloacetate decarboxylase subunit beta	10.8	4	2.757	Na+-transporting methylmalonyl-CoA/oxaloacetate decarboxylase, beta subunit		
**gi|336065500**	endonuclease/exonuclease/phosphatase family protein	11	3	1.852	Unknown		
**gi|489872418**	methionine sulfoxide reductase A	55.2	5	1.982	Peptide methionine sulfoxide reductase		
**gi|336066213**	putative metalloprotease	25.9	3	1.62	Predicted metal-dependent hydrolase		
**gi|489871071**	peptide ABC transporter ATP-binding protein	16.8	4	2.263	ABC-type antimicrobial peptide transport system, ATPase component		
**gi|336065450**	D-alanine—poly(phosphoribitol) ligase subunit 2	31.6	2	1.769	Unknown	down	ERH_0208
**gi|489871733**	ABC transporter	11.6	1	4.118	ATPase components of ABC transporters with duplicated ATPase domains		
**gi|322463931**	BadF/BadG/BcrA/BcrD ATPase family protein	31.6	7	1.671	Predicted N-acetylglucosamine kinase		
**gi|336065340**	acid phosphatase/vanadium-dependent haloperoxidase related protein	18.2	2	1.864	Uncharacterized protein conserved in bacteria	up	ERH_0098
**gi|322463944**	acetyltransferase, GNAT family	30.6	4	1.693	Histone acetyltransferase HPA2 and related acetyltransferases		
**gi|336066251**	replication initiation and membrane attachment protein	35.8	13	1.775	Replication initiation/membrane attachment protein		
**gi|322463437**	HD domain protein	24.4	8	1.55	HD superfamily phosphohydrolases		
**gi|336066440**	hypothetical protein	67.2	6	1.878	Unknown	down	ERH_1203
**gi|336065559**	N-acetyltransferase GCN5	14.6	2	1.858	Acetyltransferases, including N-acetylases of ribosomal proteins		
**gi|336066518**	hypothetical protein	10.3	1	2.093	Unknown	down	ERH_1282
**gi|489869871**	citrate lyase	34	5	1.838	Phosphoribosyl-dephospho-CoA transferase (holo-ACP synthetase)		
**gi|336065544**	YeeE/YedE family integral membrane protein	21.4	8	1.68	Predicted transporter component	up	ERH_0303
**gi|336065463**	glycoside hydrolase	23.6	11	4.251	Beta-glucanase/Beta-glucan synthetase	up	ERH_0221
**gi|336065828**	hypothetical protein	34.1	3	1.767	Unknown	down	ERH_0588
**gi|489869873**	citrate lyase subunit gamma	27.6	2	3.173	Citrate lyase, gamma subunit		
**gi|336066517**	copper chaperone	17.3	1	2.156	Copper chaperone		
**gi|322463327**	ABC 3 transport family protein	10.3	4	2.911	ABC-type Mn2+/Zn2+ transport systems, permease components		
**gi|336065783**	MarR family transcriptional regulator	17.7	2	2.505	Unknown		
**gi|336065994**	putative oxaloacetate decarboxylase subunit gamma	18	1	3.612	Transcriptional regulators		
**gi|322463480**	transcriptional regulator, LysR family	16.8	4	1.506	Unknown		
**gi|29603463**	rhusiopathiae surface protein B	4.7	3	3.364	Transcriptional regulator		
**gi|336065820**	host cell surface-exposed lipoprotein	17.6	2	1.502	Unknown		
**gi|336066586**	D-methionine ABC transporter ATP-binding protein	61.8	3	2.205	Unknown		
**gi|336065271**	hypothetical protein	51	2	1.635	ABC-type metal ion transport system, ATPase component	up	ERH_0029
**gi|322463387**	LPXTG-motif cell wall anchor domain protein	16.6	1	2.225	Uncharacterized conserved protein	up	ERH_1687
**gi|322463680**	ABC transporter, permease protein	2.3	1	17.319	Unknown	down	ERH_1245
**gi|336066678**	glycosyltransferase family protein	32.8	11	1.543	ABC-type sugar transport systems, permease components	down	ERH_1442
**gi|336065843**	hypothetical protein (Glycosyltransferases)	15.4	2	1.706	Glycosyltransferases involved in cell wall biogenesis		
**gi|336066252**	dephospho-CoA kinase	25.6	4	2.513	Predicted membrane protein		
**gi|322464309**	Biotin-requiring enzyme	19.6	2	6.095	Dephospho-CoA kinase	up	ERH_0757
**gi|322463815**	LPXTG-motif cell wall anchor domain protein	13.5	11	2.184	Biotin carboxyl carrier protein		
**gi|336065715**	hypothetical protein	18.1	5	1.712	Unknown	up	ERH_0475
**gi|322464470**	ATPase/histidine kinase/DNA gyrase B/HSP90 domain protein	35.2	12	2.296	Predicted periplasmic solute-binding protein		
**gi|322464365**	hypothetical protein HMPREF0357_10353	5.8	1	3.662	Signal transduction histidine kinase		
**gi|322463326**	ABC 3 transport family protein	8.3	3	2.436	Unknown	down	ERH_0047
**gi|336066379**	XRE family transcriptional regulator	54.4	3	2.366	ABC-type Mn2+/Zn2+ transport systems, permease components		
**gi|509078903**	ABC transporter, permease protein	6.6	2	1.617	Predicted transcriptional regulators	down	ERH_1244
**gi|336066105**	ACT domain-containing protein	30.3	3	1.705	ABC-type sugar transport system, permease component	up	ERH_0867
**gi|322464145**	putative endoribonuclease L-PSP	27.1	3	1.806	ACT domain-containing protein		
**gi|336065349**	DNA binding helix-turn helix protein	52.9	4	1.579	Putative translation initiation inhibitor, yjgF family		
**gi|336066891**	MutT/NUDIX family protein	48.6	7	3.258	Predicted transcriptional regulator		
**gi|336066864**	glycine betaine/carnitine/choline ABC transporter permease	21.1	3	8.34	NTP pyrophosphohydrolases including oxidative damage repair enzymes	up	ERH_1629
**gi|322463363**	DNA replication and repair protein RecF	9.4	3	1.701	ABC-type proline/glycine betaine transport systems, permease component	up	ERH_0007
**gi|336066579**	purine nucleosidase	50.5	13	1.543	Recombinational DNA repair ATPase (RecF pathway)		
**gi|336066735**	XRE family transcriptional regulator	42.9	5	2.366	Inosine-uridine nucleoside N-ribohydrolase		
**gi|336066556**	N-acetyltransferase GCN5	2.1	1	1.665	Predicted transcriptional regulators		
**gi|489871234**	PTS glucose transporter subunit IIBC	15.3	5	2.049	Acetyltransferases, including N-acetylases of ribosomal proteins	down	ERH_1399
**gi|336065655**	ABC transporter permease	6.1	2	1.522	Phosphotransferase system IIC components, glucose/maltose/N-acetylglucosamine-specific	up	ERH_0414
**gi|336065992**	citrate-sodium symporter	9.7	4	2.961	ABC-type sugar transport systems, permease components		
**gi|336065983**	GntR family transcriptional regulator	37.6	8	1.991	Na+/citrate symporter		
**gi|336066469**	hypothetical protein	16.9	2	1.753	Transcriptional regulators		
**gi|336066654**	MutT/NUDIX family protein	8.8	1	1.759	Unknown		
**gi|336065454**	two-component system sensor histidine kinase	25.8	8	2.469	ADP-ribose pyrophosphatase		
**gi|336066363**	ABC transporter ATP-binding protein	19.7	3	1.696	Signal transduction histidine kinase		
**gi|489870514**	beta-carotene 15,15~-monooxygenase	12.3	3	1.533	ATPase components of ABC transporters with duplicated ATPase domains	up	ERH_0064
**gi|336066325**	hypothetical protein ERH_1087	16.8	3	1.858	Uncharacterized conserved protein	up	ERH_1087
**Down-regulation proteins (67)**							
**gi|336066742**	hypothetical protein	25.5	4	0.52	Unknown		
**gi|336066589**	aspartate—ammonia ligase	32.6	9	0.407	Asparagine synthetase A	down	ERH_1353
**gi|336066753**	amino acid ABC transporter amino acid-binding protein	17.9	4	0.294	ABC-type amino acid transport/signal transduction systems, periplasmic component/domain	down	ERH_1517
**gi|336065791**	methionine adenosyltransferase	50.8	15	0.553	S-adenosylmethionine synthetase		
**gi|336065393**	putative D-alanyl-D-alanine carboxypeptidase	13.1	5	0.552	D-alanyl-D-alanine carboxypeptidase		
**gi|489869850**	bacteriocin ABC transporter ATP-binding protein	33.8	4	0.55	ABC-type antimicrobial peptide transport system, ATPase component		
**gi|336066913**	hypothetical protein	24.9	18	0.277	Unknown	down	ERH_1678
**gi|322463568**	cell envelope-like function transcriptional attenuator common domain protein	14.3	7	0.617	Transcriptional regulator		
**gi|336065737**	putative SUF system FeS cluster assembly protein SufD	37.9	8	0.637	ABC-type transport system involved in Fe-S cluster assembly, permease component		
**gi|322463946**	putative Na/Pi-cotransporter II-like protein	21.6	10	0.584	Na+/phosphate symporter		
**gi|105303396**	surface protective antigen SpaA	41.5	1	0.248	FOG: Glucan-binding domain (YG repeat)	down	ERH_0094
**gi|336065502**	subtilase familycell-envelope associated proteinase	4.5	4	0.503	Subtilisin-like serine proteases		
**gi|336065501**	cold-shock protein	74.2	4	0.503	Cold shock proteins		
**gi|336065445**	Na+ efflux pump ABC transporter permease	27	10	0.245	ABC-type Na+ efflux pump, permease component	down	ERH_0203
**gi|336066925**	guanosine monophosphate reductase 2	58.3	16	0.558	IMP dehydrogenase/GMP reductase	down	ERH_1690
**gi|336065366**	type III pantothenate kinase	22.3	4	0.571	Putative transcriptional regulator, homolog of Bvg accessory factor	down	ERH_0124
**gi|336066690**	LPXTG-motif cell wall anchor domain-containing protein	27.7	6	0.179	Unknown	down	ERH_1454
**gi|336065396**	hypothetical protein	54.7	19	0.196	Unknown	down	ERH_0154
**gi|18146962**	Tet(M)	44.6	21	0.246	Translation elongation factors (GTPases)		
**gi|322463820**	hypothetical protein HMPREF0357_11121	15.6	4	0.485	Unknown	down	ERH_1253
**gi|489869985**	hypothetical protein	8.3	1	0.493	Unknown	down	ERH_0811
**gi|322463107**	transcriptional regulator, TetR family	24.7	4	0.581	Unknown		
**gi|489871386**	dipeptidase	58.5	9	0.339	Peptidase E	down	ERH_1312
**gi|322464532**	response regulator receiver domain protein	24.6	5	0.444	Response regulators consisting of a CheY-like receiver domain and a winged-helix DNA-binding domain	down	ERH_0982
**gi|336066596**	ABC transporter permease	34.2	12	0.54	ABC-type antimicrobial peptide transport system, permease component		
**gi|336065388**	inositol monophosphatase family protein	45.5	9	0.159	Archaeal fructose-1,6-bisphosphatase and related enzymes of inositol monophosphatase family		
**gi|336066608**	ABC transporter ATP-binding protein	35.2	6	0.508	ABC-type multidrug transport system, ATPase component		
**gi|336066551**	peptidase, M42 family	46.4	11	0.536	Cellulase M and related proteins	down	ERH_1315
**gi|489871941**	pyrrolidone-carboxylate peptidase	59.2	11	0.393	Pyrrolidone-carboxylate peptidase (N-terminal pyroglutamyl peptidase)	down	ERH_0382
**gi|336066606**	enterochelin ABC transporter substrate-binding protein	39.1	9	0.48	ABC-type enterochelin transport system, periplasmic component		
**gi|336066243**	basic membrane lipoprotein	42.1	10	0.558	Uncharacterized ABC-type transport system, periplasmic component/surface lipoprotein		
**gi|336065553**	two-component system response regulator	34.2	6	0.537	Response regulators consisting of a CheY-like receiver domain and a winged-helix DNA-binding domain		
**gi|336065967**	leucine-rich repeat protein	13.5	5	0.64	Leucine-rich repeat (LRR) protein	down	ERH_0728
**gi|322463725**	PASTA domain protein	31.8	13	0.51	Unknown	down	ERH_1362
**gi|322463102**	hypothetical protein HMPREF0357_11450	7.8	1	0.531	Unknown		
**gi|503619403**	hypothetical protein	37.4	4	0.629	Unknown		
**gi|336066550**	hypothetical protein	30.6	3	0.602	Unknown		
**gi|336066068**	CobQ/CobB/MinD/ParA nucleotide binding domain-containing protein	26.9	8	0.402	Flp pilus assembly protein, ATPase CpaE		
**gi|336066221**	phosphate starvation-inducible protein PhoH	64.2	14	0.238	Phosphate starvation-inducible protein PhoH, predicted ATPase	down	ERH_0983
**gi|322463351**	PTS system glucoside-specific EIICBA component family protein	23	9	0.666	Phosphotransferase system IIC components, glucose/maltose/N-acetylglucosamine-specific		
**gi|489872217**	PTS glucose transporter subunit IIABC	15.7	6	0.208	Phosphotransferase system IIC components, glucose/maltose/N-acetylglucosamine-specific	down	ERH_0222
**gi|336065624**	hypothetical protein	8.8	2	0.467	Predicted membrane protein	down	ERH_0383
**gi|336066461**	2-dehydro-3-deoxygluconokinase	28.5	9	0.653	Sugar kinases, ribokinase family		
**gi|322464377**	Flp/Fap pilin component	14.3	1	0.304	Flp pilus assembly protein, pilin Flp	down	ERH_0825
**gi|489869981**	ABC transporter ATP-binding protein	47.4	7	0.186	ABC-type multidrug transport system, ATPase component	down	ERH_0807
**gi|336066791**	peptide chain release factor 2	40.9	11	0.634	Protein chain release factor B		
**gi|336065318**	N-acetyltransferase GCN5	22.8	5	0.642	Histone acetyltransferase HPA2 and related acetyltransferases		
**gi|336066001**	hypothetical protein	30	4	0.658	FOG: CBS domain		
**gi|489872308**	flavin reductase	37.2	5	0.628	Predicted flavoprotein		
**gi|489871309**	multidrug ABC transporter ATP-binding protein	25.2	6	0.593	ABC-type antimicrobial peptide transport system, ATPase component	down	ERH_1361
**gi|489871187**	ABC transporter	40.7	17	0.57	ABC-type multidrug transport system, ATPase and permease components		
**gi|489872323**	inositol monophosphatase	48.6	11	0.599	Archaeal fructose-1,6-bisphosphatase and related enzymes of inositol monophosphatase family		
**gi|336066429**	DNA polymerase IV	12.1	3	0.645	Nucleotidyltransferase/DNA polymerase involved in DNA repair		
**gi|322464283**	hypothetical protein HMPREF0357_10271	6.4	1	0.555	Unknown	down	ERH_0732
**gi|322463718**	ABC transporter, ATP-binding protein	3.5	1	0.497	ABC-type enterochelin transport system, ATPase component		
**gi|322463306**	hypothetical protein HMPREF0357_10606	5.1	1	0.649	Unknown		
**gi|322464381**	Flp pilus assembly protein CpaB	35.3	7	0.327	Flp pilus assembly protein CpaB		
**gi|489870985**	diacylglycerol kinase	27.1	7	0.655	Sphingosine kinase and enzymes related to eukaryotic diacylglycerol kinase		
**gi|336065298**	sulfatase family protein	31.7	15	0.446	Phosphoglycerol transferase and related proteins, alkaline phosphatase superfamily		
**gi|336066345**	alpha/beta hydrolase domain-containing protein	18.7	4	0.326	Esterase/lipase		
**gi|322464343**	ABC transporter, ATP-binding protein	11.2	6	0.659	ABC-type transport system involved in cytochrome bd biosynthesis, ATPase and permease components		
**gi|336066648**	ABC transporter permease/ATP-binding protein	23.6	14	0.644	ABC-type multidrug transport system, ATPase and permease components		
**gi|322463081**	NMT1/THI5-like protein	37.7	1	0.485	ABC-type nitrate/sulfonate/bicarbonate transport systems, periplasmic components		
**gi|336065736**	Fe-S cluster assembly ATP-binding protein	62	15	0.533	ABC-type transport system involved in Fe-S cluster assembly, ATPase component		
**gi|336066238**	peptidase M16 domain-containing protein	44.9	7	0.577	Predicted Zn-dependent peptidases		
**gi|489871578**	PTS sugar transporter	71.5	6	0.409	Phosphotransferase system, mannose/fructose-specific component IIA		
**gi|336065707**	hemolysin-like protein	28.7	10	0.396	Hemolysins and related proteins containing CBS domains		

**Fig 1:**
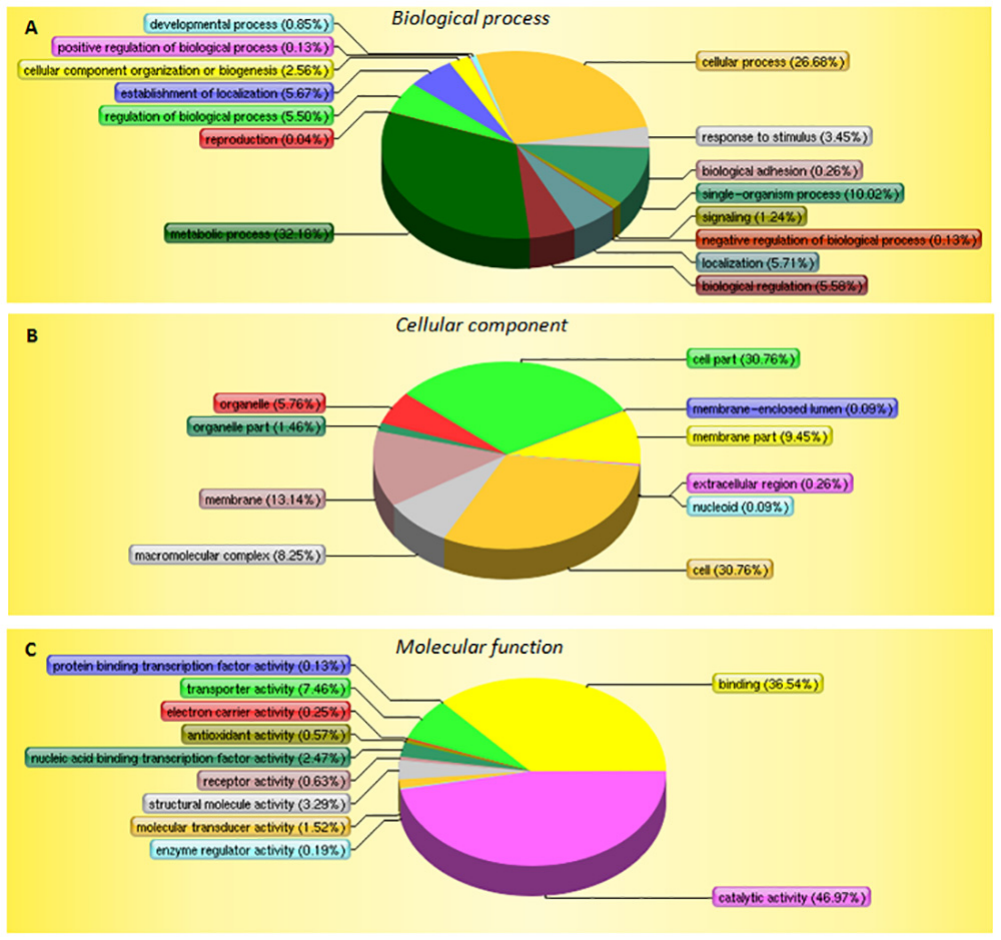
Protein classification based on functional annotations using the Gene Ontology resource. (A) GO biological processes; (B) GO molecular functions; and (C) GO cellular components. p-values were calculated using tools at the Life Science Website.

Functional annotation of the 1299 proteins was based on the three principal classifications developed by the gene ontology (http://www.geneontol.ogy.org). With respect to biological processes, most proteins were associated with metabolic processes (32.18%) and cellular processes (26.68%). When classified according to cellular component, 30.76% of proteins were described as located in the cell, 30.76% in cell parts, 13.14% in membranes, and 9.45% in membrane parts. Classification by molecular function showed that most proteins had catalytic (46.97%) and binding (36.54%) activities (Fig 1). Overall, the gene ontology classifications for the differentially expressed proteins are very similar to those obtained for all 1,299 proteins identified by Mascot. However, when the differentially expressed proteins are classified by molecular function, catalytic activity (50.75% vs. 44.07%) and binding (34.33% vs. 29.66%) are overrepresented.

**Fig 2:**
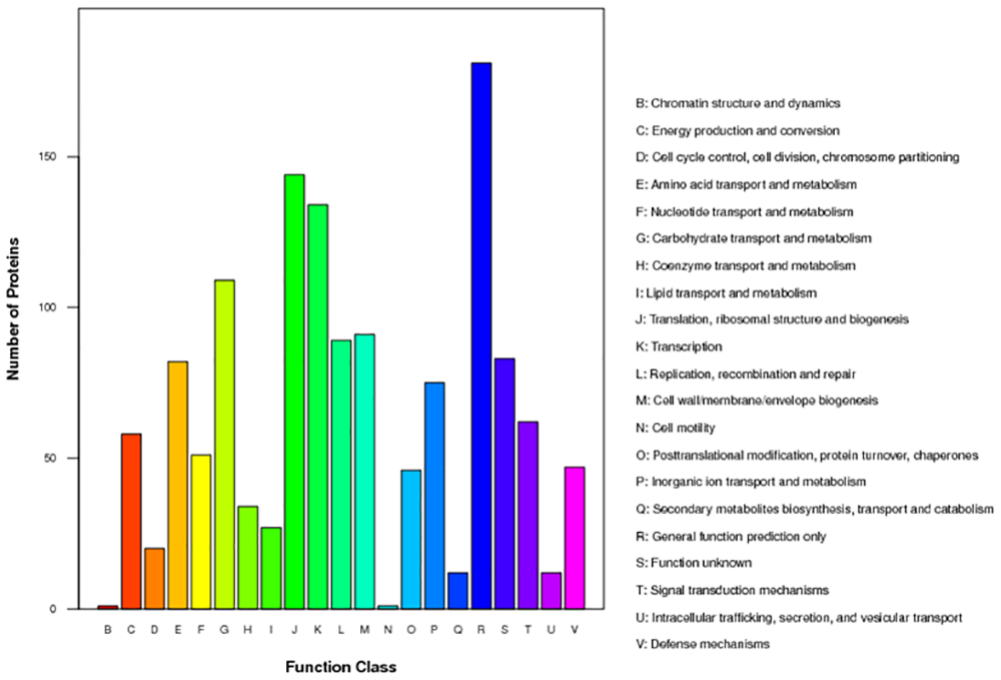
Classification of protein functions based on COGs.

The COG database (http://www.ncbi.nlm.nih.gov/COG) was used to classify and group the identified proteins (Fig 2). The ten most common categories were as follows: [R] General function prediction (~13.93%), [J] Translation, ribosomal structure and biogenesis (~11.08%), [K] Transcription (~10.31%), [G] Carbohydrate transport and metabolism (~8.39%), [M] Cell wall/membrane/envelope biogenesis (~7.00%), [L] Replication, recombination and repair (~6.85%), [S] Function unknown (~6.39%), [E] Amino acid transport and metabolism (~6.31%), [P] Inorganic ion transport and metabolism (~5.77%), and [T] Signal transduction mechanisms (~4.77%). As expected, the majority of proteins are involved in basic cellular functions, such as replication, transcription, translation and metabolism.

### Correlation between mRNA and protein expression profiles

A coupled transcriptomics-proteomics project provides a unique opportunity to investigate whether protein regulation is correlated with gene transcription. We investigated the correlation between mRNA and protein profiles and found that the levels of 1219 proteins could be correlated, either negatively or positively, with mRNA levels. Among the 168 differentially regulated proteins, only 61 could be correlated with mRNA level variations. These proteins could be clustered into four groups based on the pattern of changes in mRNA and protein levels: Group I, the mRNA and protein levels are positively correlated (45 proteins); Group II, the mRNA level remains almost unchanged while the protein level is decreased (43 proteins); Group III, the mRNA level remains almost unchanged but the protein level is increased (64 proteins); Group IV, the mRNA level is decreased but the protein level is increased (16 proteins) (Table 1). Group I includes two subgroups: both the mRNA and protein levels are increased synchronously (21 proteins); both the mRNA and protein levels are decreased synchronously (24 proteins) (Table 1).

### Protein-protein interaction analysis

**Fig 3:**
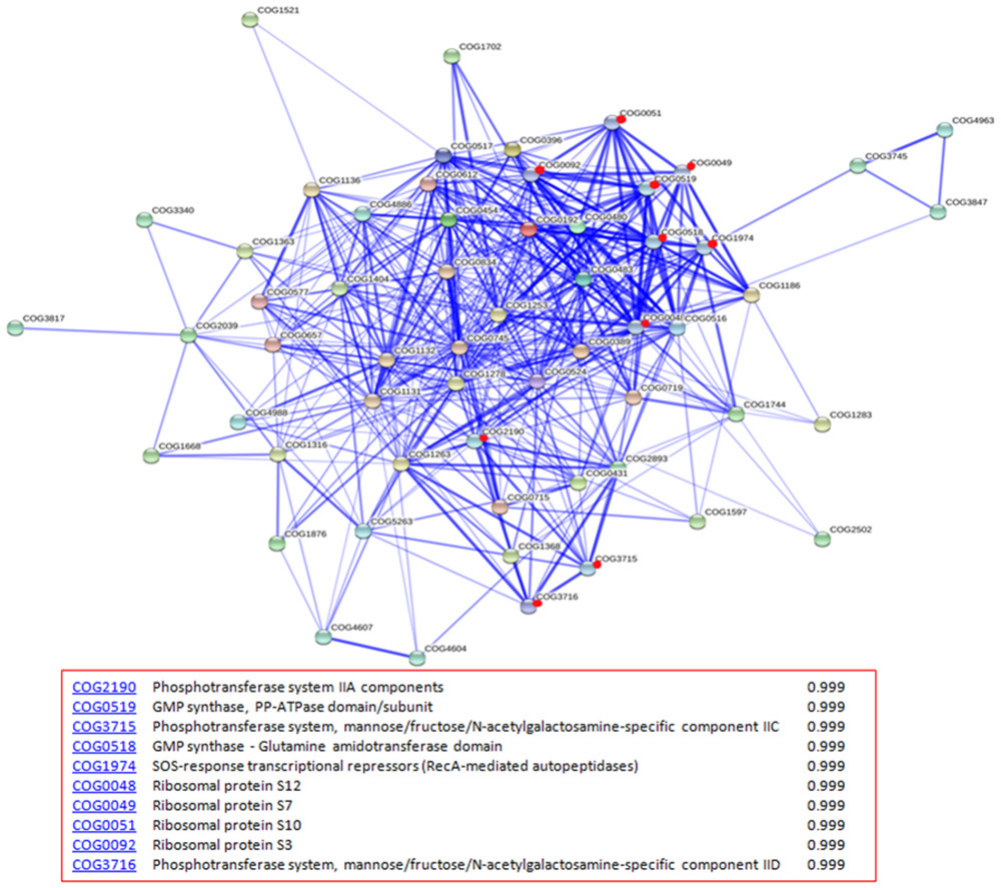
Potential protein–protein interaction network among down-regulated proteins as analyzed by STRING. Stronger associations are represented by thicker lines. Red dots indicate predicted proteins that have strong associations with the down-regulated proteins.

Protein-protein interactions play an important role in bacterial pathogenicity and metabolism. We therefore examined the 67 down-regulated proteins for potential protein interactions using the STRING database [20]. Associations were predicted to exist among these proteins, and especially between them and components of the phosphotransferase system, GMP synthases and ribosomal proteins (Fig 3). These interactions may function in reducing nucleotide and protein synthesis and saccharide phosphorylation.

### Phosphotransferase system (PTS)

**Fig 4:**
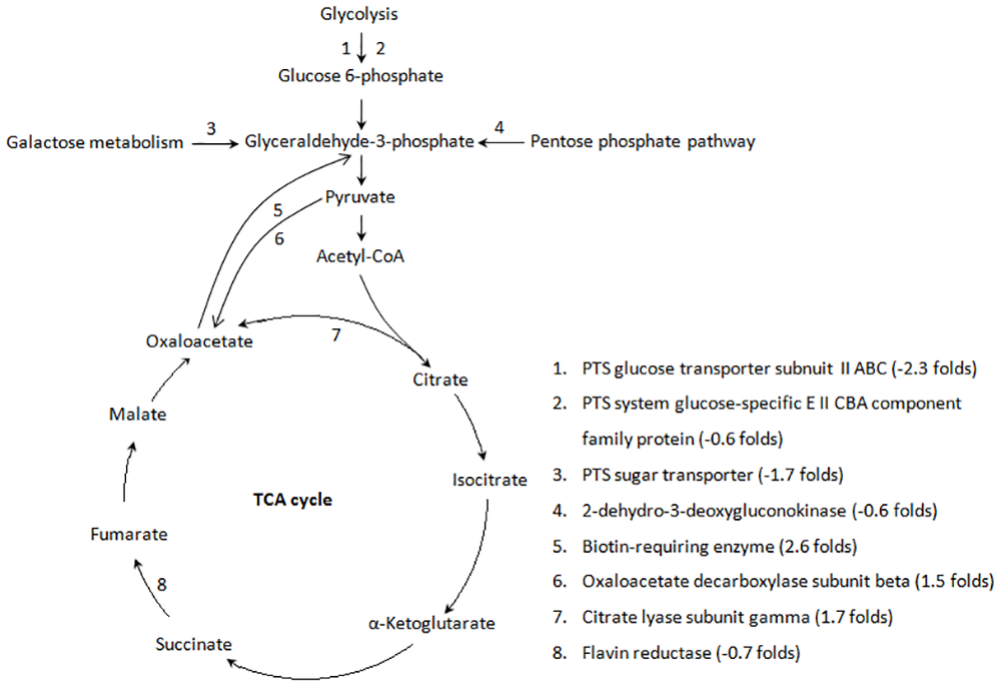
Pathway analysis of up- and down-regulated proteins associated with the TCA cycle.

The phosphoenolpyruvate sugar phosphotransferase system (PTS) is widespread among microorganisms. The PTS couples carbon source uptake and substrate phosphorylation, generating intracellular sugar-phosphate [21]. Pathway analysis suggests that the phosphorylation levels of glucose, maltose, D-glucosamine, N-acetylgalatosamine and galactosamine were down-regulated (1.6–5 fold) in HX130709a, resulting in reduced glucose 6-phosphate and glyceraldehydes-3-phophate synthesis, consistent with the up-regulation of gluconeogenesis (Fig 4).

### TCA Cycle

**Fig 5:**
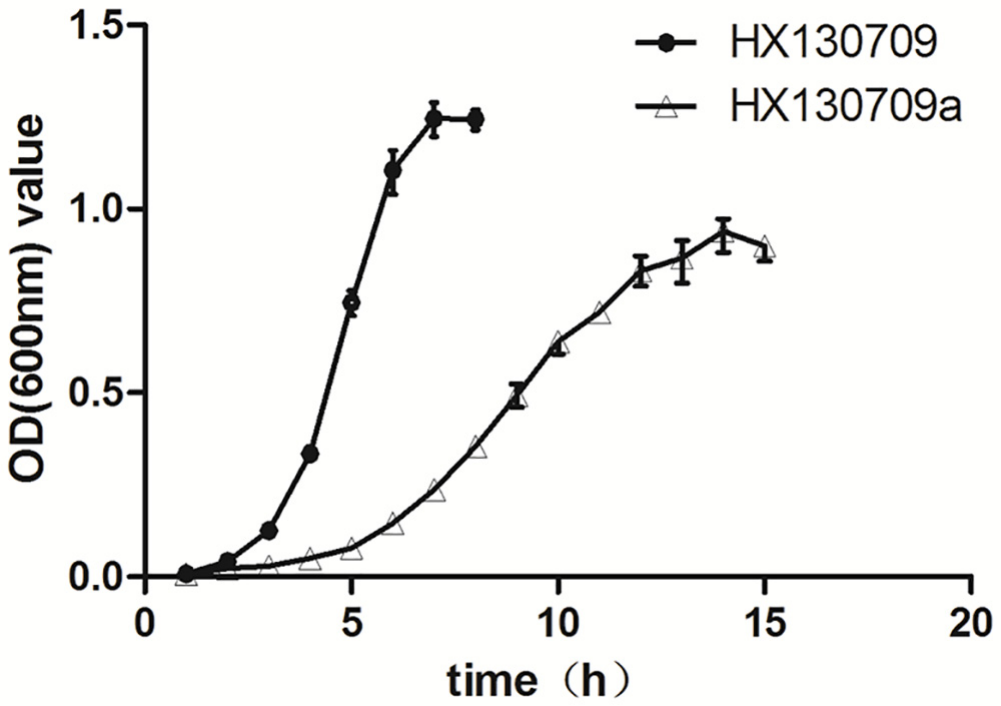
Growth curves for HX130709 and its attenuated derivative HX130907a. HX130709 and HX130709a were cultured and triplicate samples were collected at 1 h intervals. The samples were centrifuged and pellets suspended in an equal volume of PBS. Values shown are optical densities (600 nm).

**Fig 6:**
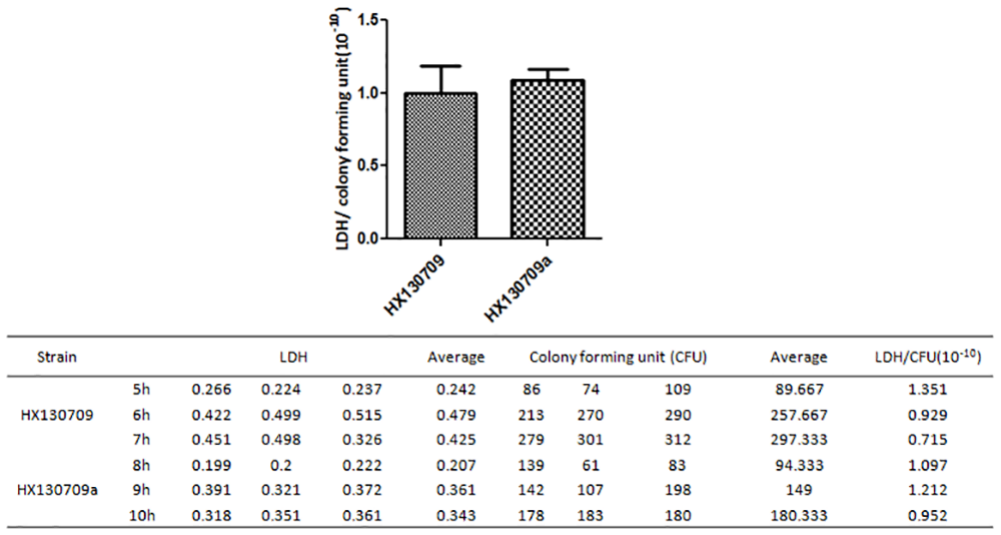
Pyruvate detection with LDH assay. Bacteria were cultured to mid-logarithmic phase in BHI medium and triplicate samples were collected at 3 time points for the virulent strain HX130709 (5 h, 6 h, 7 h) and the attenuated strain HX13079a (8 h, 9 h, 10 h). Lactate dehydrogenase levels and bacterial counts (colony forming units; CFU) were measured as described in materials and methods. Results are expressed as LDH/cfu. Pyruvate levels are assumed to correlate positively with LDH activity.

**Fig 7:**
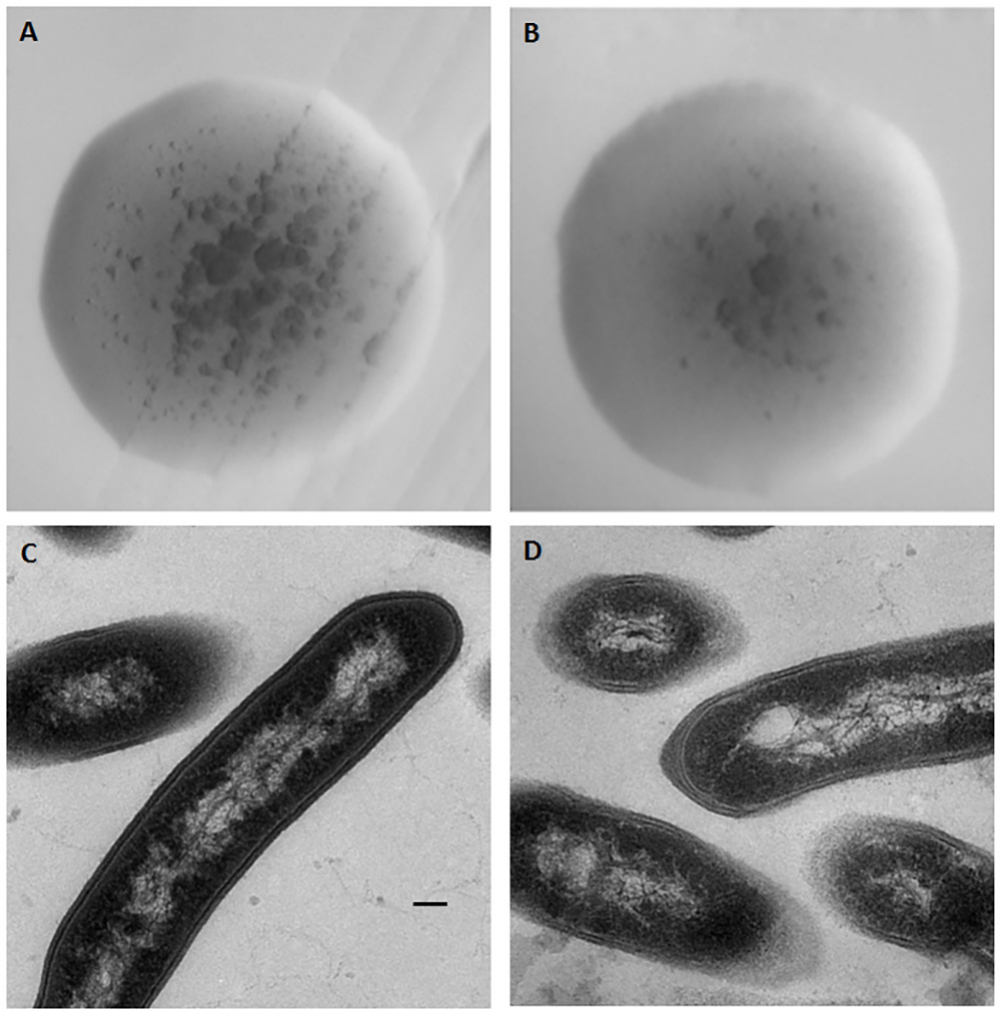
Morphological observation of HX130709 and HX130709a. Colony morphology and electron micrographs of *E*. *rhusiopathiae*. Colony morphology of (A) HX130709 and (B) HX130709a. Electron micrographs of (C) HX130709 and (D) HX130709a. The scale bar is 100 nm.

A pathway analysis focused on the TCA cycle showed that the pathway components responsible for the conversion of pyruvate and citrate to oxaloacetate were up-regulated in HX130709a relative to HX130709. In contrast, components involved in the metabolism of succinate to fumarate were down-regulated. We hypothesize that the lower levels of medium-supplied carbohydrates were used by the attenuated *E*. *rhusiopathiae*, but the cells compensated by up-regulating production of oxaloacetate and shunting excess oxaloacetate into the gluconeogenesis pathway. However, pathway components involved in the conversion of pyruvate to acetyl-CoA were not changed. Combining this fact with the down-regulation of components involved in the metabolism of succinate to fumarate, we suggest that the TCA cycle as a whole is down-regulated in HX130709a (Fig 4). Growth curves also showed that HX130709 grows more rapidly than HX130709a (Fig 5). Compared with HX130709, pyruvate levels in HX130709a may be slightly higher, but the difference is not significant (Fig 6). The colony morphology of HX130709a was convex and irregular, similar to that of HX130709. Electron microscopy showed capsule material as an electron-dense layer outside the outer membrane and no significant morphological difference between the virulent and attenuated strains (Fig 7), which is inconsistent with the result reported by Yoshihiro et al. [13, 22]. The role of the capsule in virulence has been clearly demonstrated using isogenic mutants with defined mutations. However, differences in growth curves and pyruvate metabolism between parent strains and their avirulent derivatives have not been reported, nor have differences in mRNA and protein levels. Our results demonstrate that capsule is not closely associated with virulence in *E*. *rhusiopathiae*.

### Virulence factors

In a previous study, Kwok and Li sequenced the genome of a virulent strain of *E*. *rhusiopathiae* (SY1027) and used BLASTP and VFDB to identify 37 potential virulence factors [23]. Using a similar strategy, we examined the proteins and genes identified by proteomic and transcriptomic analyses in our study and found 13 virulence factor candidates. To our surprise, compared with HX130709, no potential virulence factors were differentially expressed in HX130709a. However, it is possible that these factors are not associated with pathogenicity in *E*. *rhusiopathiae*.

Virulence factors such as neuraminidase, hyaluronidase, RspA/RspB, SpaA, and hemolysin have been suggested to play a role in *E*. *rhusiopathiae* pathogenicity. Among the proteins and genes identified in our transcriptomic and proteomic analyses, transcriptional activity was detected for neuraminidase, hyaluronidase and *spaA*, which were decreased 2.7, 2.35 and 15.8 fold in HX130709a vs. HX130709, respectively. Unexpectedly, neuraminidase was not detected as a protein product, nor were transcripts detected that corresponded to the *rspA*/*rspB* genes. Since pelleted bacteria were used in the proteomic and transcriptomic analyses, it is possible that neuraminidase was secreted into the culture medium and that the mRNAs for rspA/rspB were unstable. Protein levels for hyaluronidase and RspA were stable, but protein levels for RspB and SpaA were increased and decreased 3.364 and 4 fold, respectively. Hemolysin was not detected in mRNA or protein forms, but a hemolysin-like protein was identified and its protein level was down-regulated.

Neuraminidase is an enzyme responsible for cleavage of sialic acids from sialo-glycoconjugates such as glycoproteins, glycolipids, and oligo- and polysaccharides. The down-regulation of neuraminidase has been associated with decreased virulence in *E*. *rhusiopathiae* [10]. We found that the mRNA level of neuraminidase was decreased 2.7 fold in attenuated strain HX130907a, which is consistent with this result. Shimoji et al. conducted transposon mutagenesis with Tn916 to construct mutants defective in hyaluronidase production and reported that hyaluronidase was not associated with virulence in *E*. *rhusiopathiae* [24]. Although mRNA levels for hyaluronidase decreased 2.35 fold in our study, protein expression were essentially identical in HX130709 and HX130907a, providing additional support that hyaluronidase is not associated with virulence in *E*. *rhusiopathiae*. RspA expression was also identical in the two strains, but RspB was increased in the attenuated strain HX130907a, suggesting that RspA and RspB are not associated with virulence in *E*. *rhusiopathiae*. Finally, Borrathybay et al. deleted the *spaA* gene from wild-type *E*. *rhusiopathiae* strain C43065 and found that the virulence of the Δ*spaA* mutant decreased more than 76 fold [25], but they did not compare growth curves for the wild-type and mutant strains. We also found that *spaA* transcription and SpaA protein levels decreased in HX130907a, supporting the hypothesis that SpaA is associated with virulence in *E*. *rhusiopathiae*.

In summary, the transcriptomes and proteomes of an attenuated *E*. *rhusiopathiae* and its parent strain were compared. 475 genes and 168 proteins were found to be up- or down-regulated and the levels for 61 proteins could be correlated with gene transcription levels. The growth of the attenuated strain is slower than its parent strain, but pyruvate metabolism appears to be unaffected. Our data are consistent with other studies showing that SpaA and neuraminidase, but not hyaluronidase and capsule, are associated with virulence in *E*. *rhusiopathiae*. We conclude that the down-regulation of the TCA cycle and the down-regulation of several proteins are associated with virulence in this organism.

## Supporting Information

S1 Fig Comparisons between various biological replicates. The difference was plotted versus the percentage of the proteins identified. Approximately 82.4% of proteins had cv differences less than 0.1, and more than 99.7% of the proteins had cv errors less than 0.5.
(PNG)

S2 Fig Survival rate of mice in each group after infection. (TIF)

S3 Fig Antibody levels in serum of mice immunized with different type of vaccines. (TIF)

S1 File Full annotation of proteins identified in strains HX130709a and HX130709. (XLS)

S1 Table Differentially regulated genes identified by RNA-Seq (HX130709a/HX130709). (DOCX)
