# Supplementary figures and images for: Proteomic and Transcriptomic Analyses of Swine Pathogen Erysipelothrix rhusiopathiae Reveal Virulence Repertoire
Source: PLoS One. 2016 Aug 1;11(8):e0159462. doi: 10.1371/journal.pone.0159462 (PMC4968806; doi:10.1371/journal.pone.0159462)

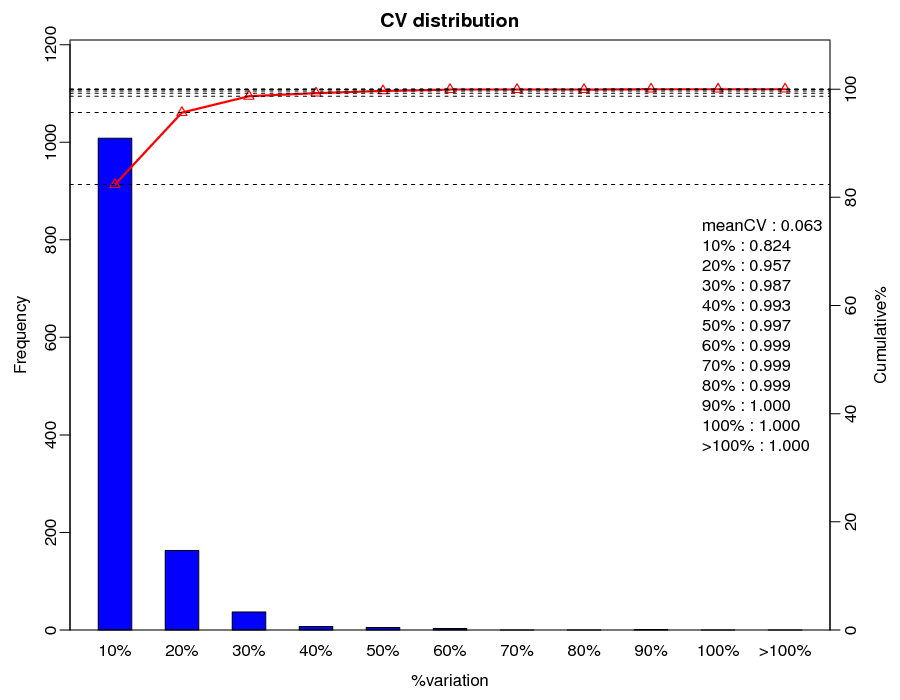

Supplement: S1 Fig — The difference was plotted versus the percentage of the proteins identified. Approximately 82.4% of proteins had cv differences less than 0.1, and more than 99.7% of the proteins had cv errors less than 0.5. (PNG) [file pone.0159462.s001.png]

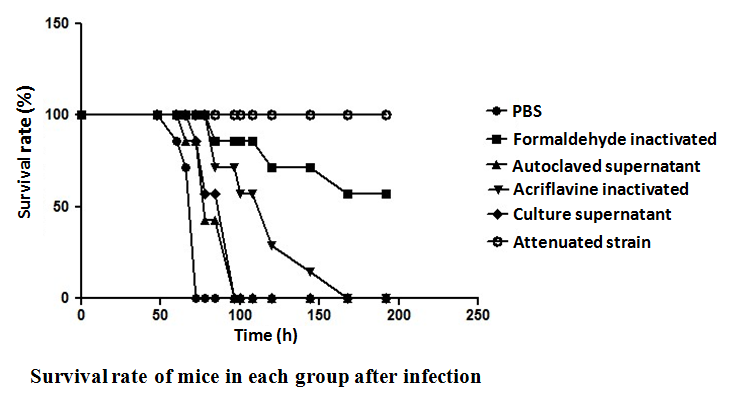

Supplement: S2 Fig — (TIF) [file pone.0159462.s002.tif]

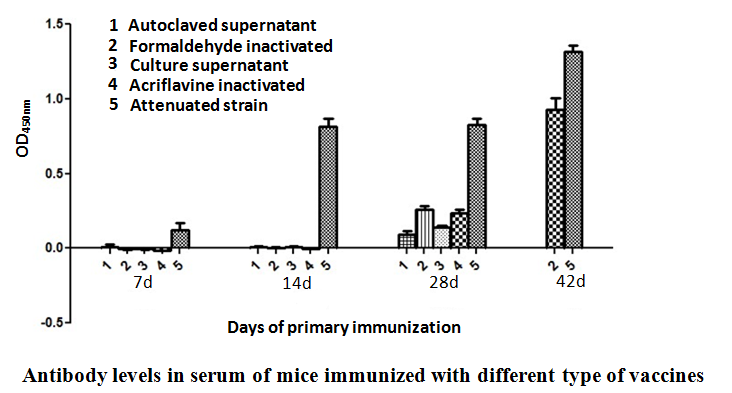

Supplement: S3 Fig — (TIF) [file pone.0159462.s003.tif]
