# Supplementary material for: Proteomic and Transcriptomic Analyses of Swine Pathogen Erysipelothrix rhusiopathiae Reveal Virulence Repertoire
Source: PLoS One. 2016 Aug 1;11(8):e0159462. doi: 10.1371/journal.pone.0159462 (PMC4968806; doi:10.1371/journal.pone.0159462)
Supplement: S1 Table — (DOCX) [file pone.0159462.s005.docx]

Supplemental Table 1. Differentially regulated genes identified in transcriptome (HX130709a/HX130709).

| geneID | geneLength | HX130709-RPKM | HX130709a-RPKM | log2 Ratio | Regulation | P-value | Product |
| --- | --- | --- | --- | --- | --- | --- | --- |
| ERH_1401 | 2583 | 543.9710893 | 9497.777429 | 4.125988 | Up | 1.93E-11 | calcium-translocating P-type ATPase; |
| ERH_1630 | 1131 | 69.10028337 | 773.571644 | 3.484771 | Up | 4.99E-13 | glycine betaine/carnitine/choline ABC transporter ATP-binding protein; |
| ERH_0519 | 510 | 116.7419927 | 1267.13278 | 3.440172 | Up | 2.11E-13 | hypothetical protein; |
| ERH_1629 | 642 | 46.78006761 | 448.0088536 | 3.259561 | Up | 0 | glycine betaine/carnitine/choline ABC transporter permease; |
| ERH_0219 | 1281 | 76.99510262 | 647.2861071 | 3.071565 | Up | 1.41E-13 | PTS system cellobiose-specific transporter subunit IIC; |
| ERH_1059 | 420 | 77.72408195 | 581.6581943 | 2.903738 | Up | 6.84E-14 | ATP-binding protein; |
| ERH_1438 | 1044 | 1024.456877 | 7658.07217 | 2.902122 | Up | 1.67E-11 | auxin efflux carrier family protein; |
| ERH_0221 | 2100 | 108.8387651 | 785.4525977 | 2.851332 | Up | 0 | glycoside hydrolase; |
| ERH_0889 | 405 | 58.13074549 | 416.1664989 | 2.839788 | Up | 0 | transcription antitermination protein NusB; |
| ERH_1094 | 660 | 38.90656913 | 271.3786307 | 2.802221 | Up | 0 | hypothetical protein; |
| ERH_1150 | 219 | 115.3986498 | 764.1575663 | 2.727244 | Up | 0 | translation initiation factor IF-1; |
| ERH_1627 | 894 | 71.77342227 | 430.6833635 | 2.585106 | Up | 0 | glycine betaine/carnitine/choline ABC transporter substrate-binding protein; |
| ERH_0567 | 1257 | 65.60875904 | 373.9122134 | 2.510739 | Up | 0 | FtsW/RodA/SpoVE family cell division protein; |
| ERH_0821 | 1056 | 34.26249204 | 187.3800249 | 2.451265 | Up | 1.95E-13 | VanZ family protein; |
| ERH_1628 | 606 | 62.24356375 | 328.2307984 | 2.398714 | Up | 2.71E-14 | glycine betaine/carnitine/choline ABC transporter permease; |
| ERH_0220 | 900 | 135.477515 | 706.9800065 | 2.383616 | Up | 0 | hypothetical protein; |
| ERH_1207 | 330 | 14.04842067 | 72.39717054 | 2.365525 | Up | 8.54E-10 | phnA protein; |
| ERH_0413 | 1071 | 286.7244229 | 1451.521579 | 2.339829 | Up | 1.33E-12 | ABC transporter ATP-binding protein; |
| ERH_0741 | 1047 | 973.1811607 | 4911.482737 | 2.335378 | Up | 1.24E-11 | putative regulatory protein; |
| ERH_1600 | 540 | 40.07190386 | 198.0277119 | 2.305039 | Up | 0 | hypoxanthine phosphoribosyltransferase; |
| ERH_0972 | 897 | 177.6001501 | 836.9175636 | 2.236453 | Up | 2.68E-13 | GTP-binding protein Era; |
| ERH_1057 | 429 | 33.77520644 | 156.6065575 | 2.213108 | Up | 0 | ribosomal-protein-alanine N-acetyltransferase; |
| ERH_1056 | 1011 | 208.7824041 | 935.5780042 | 2.163858 | Up | 0 | O-sialoglycoprotein endopeptidase; |
| ERH_1158 | 399 | 402.9718394 | 1775.540633 | 2.139507 | Up | 4.65E-13 | 30S ribosomal protein S8; |
| ERH_0645 | 651 | 109.8660383 | 468.9856366 | 2.093798 | Up | 3.32E-13 | di-trans,poly-cis-decaprenylcistransferase; |
| ERH_0303 | 1263 | 477.5290065 | 2001.676836 | 2.067549 | Up | 0 | YeeE/YedE family integral membrane protein; |
| ERH_1443 | 186 | 18.49809055 | 77.44206419 | 2.065741 | Up | 5.27E-08 | hypothetical protein; |
| ERH_1661 | 558 | 103.2162858 | 430.4436045 | 2.060154 | Up | 6.32E-13 | primase-like protein; |
| ERH_0553 | 633 | 324.2185726 | 1312.429931 | 2.017202 | Up | 2.47E-12 | tRNA (guanine-N(7)-)-methyltransferase; |
| ERH_0641 | 924 | 1988.089481 | 7975.643632 | 2.004218 | Up | 0 | 30S ribosomal protein S2; |
| ERH_0894 | 834 | 53.02557997 | 212.422933 | 2.002179 | Up | 1.89E-13 | tRNA pseudouridine synthase B; |
| ERH_1159 | 270 | 935.0874128 | 3726.298707 | 1.99457 | Up | 3.99E-12 | 30S ribosomal protein S14; |
| ERH_1173 | 312 | 445.0238688 | 1742.422249 | 1.96914 | Up | 6.37E-13 | 30S ribosomal protein S10; |
| ERH_0928 | 525 | 4170.027161 | 16042.8306 | 1.9438 | Up | 0 | hypothetical protein; |
| ERH_0074 | 1857 | 510.0848668 | 1954.369786 | 1.937894 | Up | 1.07E-11 | heavy metal-translocating P-type ATPase; |
| ERH_1157 | 546 | 649.9946281 | 2468.833403 | 1.92533 | Up | 4.07E-12 | 50S ribosomal protein L6; |
| ERH_0520 | 147 | 43.15907183 | 163.8997139 | 1.925078 | Up | 9.70E-12 | 50S ribosomal protein L32; |
| ERH_1020 | 840 | 110.6374677 | 418.3740337 | 1.918953 | Up | 3.16E-13 | rod shape-determining protein MreC; |
| ERH_0770 | 1191 | 209.6686737 | 788.8514918 | 1.911642 | Up | 1.53E-12 | F420-0:gamma-glutamyl ligase-like protein; |
| ERH_0953 | 663 | 100.5909444 | 375.5710523 | 1.900585 | Up | 3.48E-13 | cytidylate kinase; |
| ERH_0666 | 1659 | 300.7759254 | 1089.243231 | 1.856565 | Up | 0 | RNA-metabolising metallo-beta-lactamase; |
| ERH_1118 | 483 | 63.67091331 | 229.1173522 | 1.84738 | Up | 1.22E-13 | pantetheine-phosphate adenylyltransferase; |
| ERH_0938 | 603 | 55.46524252 | 197.4437123 | 1.831786 | Up | 0 | glycerol-3-phosphate acyltransferase PlsY; |
| ERH_0406 | 654 | 146.7318591 | 520.4180608 | 1.826489 | Up | 0 | sugar transferase; |
| ERH_0800 | 321 | 30.21018175 | 106.248373 | 1.814334 | Up | 5.57E-14 | hypothetical protein; |
| ERH_0855 | 1329 | 111.3013533 | 389.9671362 | 1.808881 | Up | 0 | exopolysaccharide biosynthesis polyprenyl glycosylphosphotransferase; |
| ERH_1651 | 1755 | 361.7192501 | 1263.685826 | 1.804696 | Up | 0 | ATP-dependent RNA helicase DeaD; |
| ERH_1164 | 204 | 3082.44367 | 10759.02524 | 1.803401 | Up | 0 | 50S ribosomal protein L29; |
| ERH_1106 | 462 | 136.6602695 | 471.221204 | 1.785811 | Up | 0 | signal peptidase II; |
| ERH_0661 | 954 | 172.2905823 | 591.6126555 | 1.779809 | Up | 8.39E-14 | XRE family transcriptional regulator; |
| ERH_0194 | 351 | 24.66465616 | 84.2869105 | 1.772863 | Up | 2.29E-12 | putative LrgA family protein; |
| ERH_1676 | 756 | 179.6858626 | 609.3137144 | 1.761708 | Up | 0 | ParB-like partition protein; |
| ERH_0665 | 765 | 72.16966663 | 243.752071 | 1.75595 | Up | 1.96E-13 | metallophosphoesterase; |
| ERH_0262 | 594 | 67.35506829 | 226.3602497 | 1.748762 | Up | 1.37E-13 | peptidase propeptide and ypeb domain-containing protein; |
| ERH_0742 | 996 | 1914.805715 | 6430.168531 | 1.747659 | Up | 0 | D-specific D-2-hydroxyacid dehydrogenase; |
| ERH_0491 | 2139 | 355.5929436 | 1191.971115 | 1.745051 | Up | 0 | polyribonucleotide nucleotidyltransferase; |
| ERH_0474 | 342 | 1105.708531 | 3671.409299 | 1.731363 | Up | 2.56E-12 | hypothetical protein; |
| ERH_1253 | 828 | 117.8969191 | 389.3127771 | 1.723404 | Up | 0 | hypothetical protein; |
| ERH_0028 | 870 | 1494.697273 | 4929.618578 | 1.721623 | Up | 2.40E-11 | DegV family protein; |
| ERH_1328 | 1029 | 223.853939 | 737.5713269 | 1.720225 | Up | 1.94E-12 | dihydrodipicolinate reductase domain-containing protein; |
| ERH_0939 | 717 | 160.240028 | 510.2330724 | 1.670922 | Up | 0 | ABC transporter ATP-binding protein; |
| ERH_0475 | 1083 | 170.2032972 | 540.9487416 | 1.668233 | Up | 3.99E-13 | hypothetical protein; |
| ERH_1037 | 552 | 57.64860303 | 181.5182864 | 1.654757 | Up | 0 | hypothetical protein; |
| ERH_0985 | 1428 | 52.70350486 | 165.5710033 | 1.651479 | Up | 0 | ATP-dependent RNA helicase RhlE; |
| ERH_1639 | 1179 | 54.74767962 | 168.9702303 | 1.625899 | Up | 0 | acetyl-CoA C-acetyltransferase; |
| ERH_0007 | 1086 | 40.97805614 | 125.7538667 | 1.617679 | Up | 1.89E-13 | DNA replication and repair protein RecF; |
| ERH_0166 | 390 | 42.71239376 | 130.357219 | 1.609744 | Up | 0 | hypothetical protein; |
| ERH_0470 | 621 | 63.90355616 | 194.755814 | 1.607698 | Up | 1.25E-13 | fused transcriptional regulator, GntR family/TrkA-C domain-containing protein; |
| ERH_0974 | 369 | 103.6766796 | 312.6801875 | 1.592596 | Up | 0 | diacylglycerol kinase; |
| ERH_1087 | 522 | 58.55643226 | 172.3667966 | 1.557582 | Up | 9.64E-14 | hypothetical protein; |
| ERH_1528 | 537 | 1664.470347 | 4899.084848 | 1.557449 | Up | 1.78E-11 | transcription antitermination protein NusG; |
| ERH_1178 | 420 | 276.49106 | 803.0687017 | 1.538291 | Up | 0 | 30S ribosomal protein S12; |
| ERH_1172 | 693 | 1054.408597 | 3048.088443 | 1.531471 | Up | 0 | 50S ribosomal protein L3; |
| ERH_1180 | 522 | 24.68711323 | 71.1470879 | 1.527047 | Up | 2.41E-13 | thermonuclease; |
| ERH_1112 | 699 | 69.65991665 | 200.5718339 | 1.525718 | Up | 0 | ABC transporter ATP-binding protein; |
| ERH_1131 | 408 | 3639.487005 | 10416.00176 | 1.516995 | Up | 2.63E-11 | 30S ribosomal protein S9; |
| ERH_1041 | 1704 | 1256.971921 | 3552.811667 | 1.499009 | Up | 7.97E-12 | phosphoenolpyruvate-protein phosphotransferase; |
| ERH_0893 | 939 | 124.4133939 | 350.3024266 | 1.493459 | Up | 3.78E-13 | riboflavin kinase/FAD synthetase; |
| ERH_0367 | 513 | 293.2653362 | 820.7190836 | 1.484682 | Up | 0 | ATP synthase F0 subunit B; |
| ERH_1025 | 192 | 85.89585672 | 239.4717368 | 1.479195 | Up | 0 | 50S ribosomal protein L28; |
| ERH_0648 | 444 | 374.0325437 | 1040.431551 | 1.475946 | Up | 0 | hypothetical protein; |
| ERH_1687 | 4377 | 57.22194948 | 159.1484095 | 1.475732 | Up | 4.97E-12 | surface protein C; |
| ERH_1053 | 1299 | 53.78964113 | 149.4076342 | 1.473854 | Up | 0 | N-acetylmuramoyl-L-alanine amidase; |
| ERH_1160 | 546 | 800.4394017 | 2209.630937 | 1.464941 | Up | 0 | 50S ribosomal protein L5; |
| ERH_1117 | 1527 | 107.1323634 | 295.3675048 | 1.463117 | Up | 2.91E-13 | integral membrane protein MviN; |
| ERH_0098 | 465 | 40.25502283 | 110.0970797 | 1.451535 | Up | 0 | acid phosphatase/vanadium-dependent haloperoxidase related protein; |
| ERH_0006 | 213 | 49.40633932 | 134.8714482 | 1.448817 | Up | 7.89E-10 | S4-like RNA-binding protein; |
| ERH_0956 | 558 | 325.7420441 | 888.8629657 | 1.448231 | Up | 1.17E-13 | translation elongation factor P; |
| ERH_0448 | 441 | 33.29267394 | 90.39516286 | 1.441041 | Up | 1.21E-12 | MarR family transcriptional regulator; |
| ERH_1670 | 1341 | 196.4387219 | 531.1332791 | 1.434995 | Up | 3.98E-12 | tRNA modification GTPase TrmE; |
| ERH_0414 | 885 | 193.8812142 | 519.804033 | 1.422795 | Up | 5.22E-13 | ABC transporter permease; |
| ERH_0757 | 324 | 92.91548593 | 248.0228905 | 1.416482 | Up | 1.03E-13 | acetyl-CoA carboxylase, biotin carboxyl carrier protein; |
| ERH_0512 | 561 | 135.4811277 | 360.5290559 | 1.412024 | Up | 0 | signal peptidase I; |
| ERH_0526 | 912 | 199.0623213 | 527.8107092 | 1.4068 | Up | 0 | S-adenosyl-L-methionine-dependent methyltransferase, MraW; |
| ERH_0097 | 240 | 73.59700326 | 194.9130794 | 1.405112 | Up | 0 | NifU-like domain-containing protein; |
| ERH_0595 | 624 | 38.94661538 | 102.0549541 | 1.389776 | Up | 2.00E-14 | hypothetical protein; |
| ERH_1677 | 717 | 224.8663558 | 586.8707707 | 1.383975 | Up | 3.91E-13 | ribosomal RNA small subunit methyltransferase G; |
| ERH_1063 | 489 | 115.3015266 | 300.4188979 | 1.381564 | Up | 4.05E-13 | phosphatidylglycerophosphatase A; |
| ERH_0652 | 462 | 28.41104632 | 73.89190369 | 1.378964 | Up | 6.47E-11 | hypothetical protein; |
| ERH_0029 | 627 | 109.8233558 | 285.4293878 | 1.377949 | Up | 0 | hypothetical protein; |
| ERH_1503 | 711 | 83.07996824 | 215.2276741 | 1.373291 | Up | 1.64E-13 | hypothetical protein; |
| ERH_0366 | 243 | 213.4252381 | 546.7008301 | 1.357021 | Up | 0 | ATP synthase F0 subunit C; |
| ERH_1054 | 1383 | 851.8840014 | 2171.683216 | 1.350085 | Up | 1.10E-11 | asparaginyl-tRNA synthetase; |
| ERH_1236 | 447 | 98.27409991 | 249.8857882 | 1.346386 | Up | 5.95E-14 | hypothetical protein; |
| ERH_0846 | 1584 | 135.8801028 | 343.0370318 | 1.33603 | Up | 1.20E-12 | ABC transporter ATP-binding protein; |
| ERH_0964 | 150 | 127.5387068 | 321.2989005 | 1.332981 | Up | 0 | 50S ribosomal protein L33; |
| ERH_0644 | 546 | 426.7594032 | 1070.223539 | 1.326417 | Up | 0 | ribosome recycling factor; |
| ERH_0973 | 402 | 282.5688069 | 707.187233 | 1.32349 | Up | 0 | cytidine deaminase; |
| ERH_1311 | 879 | 109.1483364 | 272.4697995 | 1.319806 | Up | 9.10E-15 | thioredoxin-disulfide reductase; |
| ERH_1385 | 933 | 1681.132489 | 4164.677205 | 1.308771 | Up | 0 | alpha/beta fold family hydrolase; |
| ERH_0108 | 228 | 249.409016 | 615.0541181 | 1.3022 | Up | 5.24E-14 | preprotein translocase subunit SecG; |
| ERH_0218 | 1179 | 67.8377551 | 167.2414265 | 1.301772 | Up | 0 | ROK family protein; |
| ERH_0642 | 885 | 1788.406491 | 4379.11255 | 1.291964 | Up | 4.17E-12 | translation elongation factor Ts; |
| ERH_0365 | 675 | 224.8566701 | 549.4945751 | 1.2891 | Up | 3.36E-13 | ATP synthase F0 subunit A; |
| ERH_0984 | 186 | 3551.896717 | 8675.543447 | 1.288365 | Up | 0 | 30S ribosomal protein S21; |
| ERH_1040 | 657 | 18.35314887 | 44.82075573 | 1.288139 | Up | 1.72E-08 | HD superfamily phosphohydrolase; |
| ERH_0971 | 699 | 47.19558558 | 114.4540955 | 1.278045 | Up | 1.24E-13 | DNA repair protein RecO; |
| ERH_0954 | 978 | 246.3588044 | 596.1173458 | 1.274835 | Up | 2.55E-12 | hypothetical protein; |
| ERH_0662 | 594 | 234.9089001 | 567.2126187 | 1.271788 | Up | 0 | CDP-diacylglycerol--glycerol-3-phosphate 3-phosphatidyltransferase; |
| ERH_1289 | 1908 | 42.21073039 | 101.1892484 | 1.261374 | Up | 1.31E-13 | N-6 adenine-specific DNA methylase; |
| ERH_0305 | 1308 | 101.4106872 | 242.8589502 | 1.259909 | Up | 0 | amino acid permease family protein; |
| ERH_1163 | 261 | 2265.689467 | 5415.500776 | 1.257145 | Up | 0 | 30S ribosomal protein S17; |
| ERH_0675 | 1119 | 347.2822474 | 827.7101721 | 1.253017 | Up | 2.06E-12 | tRNA (5-methyl aminomethyl-2-thiouridylate)-methyltransferase; |
| ERH_1058 | 564 | 138.3785747 | 328.5265684 | 1.247389 | Up | 0 | peptidase, M22 family; |
| ERH_1105 | 909 | 349.3453719 | 826.4474599 | 1.242269 | Up | 6.36E-13 | ribosomal large subunit pseudouridine synthase D; |
| ERH_0050 | 618 | 44.58332023 | 104.7864584 | 1.232876 | Up | 4.15E-14 | phosphatase; |
| ERH_0013 | 624 | 294.3129244 | 690.9248633 | 1.231178 | Up | 0 | sortase family protein; |
| ERH_0525 | 453 | 112.6639354 | 264.464776 | 1.23105 | Up | 5.08E-14 | cell division protein MraZ; |
| ERH_0271 | 564 | 152.4728564 | 356.4028377 | 1.224956 | Up | 0 | accessory gene regulator B; |
| ERH_0546 | 720 | 89.63313883 | 208.1908603 | 1.215803 | Up | 0 | integral membrane protein; |
| ERH_1631 | 753 | 91.47263105 | 212.3097454 | 1.214759 | Up | 2.12E-13 | formate/nitrite transporter family protein; |
| ERH_0385 | 1233 | 748.390042 | 1730.153908 | 1.209038 | Up | 1.29E-11 | hypothetical protein; |
| ERH_1069 | 483 | 2074.011255 | 4790.678449 | 1.207806 | Up | 2.01E-11 | DivIVA family protein; |
| ERH_1177 | 471 | 975.550925 | 2243.552005 | 1.201496 | Up | 0 | 30S ribosomal protein S7; |
| ERH_0653 | 507 | 57.81694715 | 132.9604516 | 1.201433 | Up | 1.05E-13 | DNA replication protein DnaD; |
| ERH_0913 | 588 | 55.54259996 | 127.6702406 | 1.200756 | Up | 1.44E-13 | Holliday junction ATP-dependent DNA helicase RuvA; |
| ERH_0176 | 558 | 681.1075164 | 1564.049639 | 1.199332 | Up | 2.79E-12 | thioredoxin-like fold-containing protein; |
| ERH_1290 | 579 | 75.46669732 | 172.5511368 | 1.193112 | Up | 0 | XRE family transcriptional regulator; |
| ERH_0353 | 369 | 52.15294 | 119.1234111 | 1.191636 | Up | 2.77E-10 | ribonucleotide reductase stimulatory protein; |
| ERH_1132 | 438 | 4139.711979 | 9355.989565 | 1.17636 | Up | 0 | 50S ribosomal protein L13; |
| ERH_0368 | 540 | 458.5689773 | 1036.303247 | 1.176236 | Up | 1.45E-12 | ATP synthase F1 subunit delta; |
| ERH_1016 | 2502 | 143.5284766 | 323.7405072 | 1.173501 | Up | 3.99E-12 | DNA polymerase I; |
| ERH_0304 | 1698 | 1772.415048 | 3989.90594 | 1.170638 | Up | 0 | pyridine nucleotide-disulfide oxidoreductase family protein; |
| ERH_0654 | 621 | 83.36492384 | 187.5142527 | 1.169488 | Up | 0 | endonuclease III; |
| ERH_1119 | 423 | 32.66083104 | 73.32479977 | 1.16674 | Up | 1.53E-07 | flavodoxin; |
| ERH_1111 | 1584 | 57.67529563 | 129.2600265 | 1.164251 | Up | 0 | hypothetical protein; |
| ERH_1532 | 375 | 121.3334382 | 271.2073641 | 1.160419 | Up | 5.39E-13 | ribonuclease III family protein; |
| ERH_1229 | 594 | 56.13607819 | 125.4408198 | 1.160007 | Up | 0 | hypothetical protein; |
| ERH_0386 | 1080 | 70.44407991 | 156.9455734 | 1.155714 | Up | 3.33E-13 | hypothetical protein; |
| ERH_0782 | 735 | 124.1378434 | 276.4224859 | 1.154932 | Up | 0 | hypothetical protein; |
| ERH_0528 | 2181 | 225.1566597 | 496.8454033 | 1.141868 | Up | 0 | penicillin-binding protein 2B; |
| ERH_0263 | 531 | 72.28737092 | 158.784063 | 1.135251 | Up | 2.71E-14 | dihydrofolate reductase family protein; |
| ERH_0844 | 1242 | 48.27407919 | 105.7454957 | 1.131276 | Up | 1.63E-13 | competence/damage-inducible protein CinA; |
| ERH_0027 | 855 | 403.3030711 | 877.8494754 | 1.122109 | Up | 3.69E-12 | DegV family protein; |
| ERH_1194 | 729 | 124.1009979 | 269.6561412 | 1.119606 | Up | 0 | two-component system response regulator LytTR family; |
| ERH_0547 | 513 | 92.7511418 | 201.2460608 | 1.117524 | Up | 0 | RNA methyltransferase; |
| ERH_0682 | 669 | 234.4173828 | 508.241692 | 1.116435 | Up | 0 | hypothetical protein; |
| ERH_0837 | 378 | 25.53130052 | 55.35146785 | 1.116355 | Up | 2.20E-05 | cytidine/deoxycytidylate deaminase family protein; |
| ERH_0344 | 1209 | 137.7774543 | 297.7856734 | 1.111935 | Up | 0 | putative O-antigen polymerase family protein; |
| ERH_1400 | 849 | 165.0536414 | 356.5252643 | 1.111069 | Up | 0 | transcriptional antiterminator BglG; |
| ERH_0032 | 1113 | 75.4943304 | 162.8581671 | 1.109176 | Up | 0 | cation efflux family protein; |
| ERH_0801 | 969 | 25.92683491 | 55.48709392 | 1.097706 | Up | 4.20E-11 | hypothetical protein; |
| ERH_0935 | 1305 | 300.5034985 | 636.9808616 | 1.083868 | Up | 1.12E-12 | adenylosuccinate lyase; |
| ERH_1446 | 648 | 167.4340765 | 354.2605202 | 1.081218 | Up | 0 | sugar transferase; |
| ERH_0921 | 669 | 1299.445177 | 2740.951603 | 1.076781 | Up | 1.86E-11 | two-component system response regulator; |
| ERH_1225 | 795 | 37.13926404 | 78.2112934 | 1.074432 | Up | 2.36E-12 | gluconate 5-dehydrogenase; |
| ERH_0739 | 984 | 46.27760668 | 97.27410861 | 1.071742 | Up | 1.42E-14 | tRNA-dihydrouridine synthase; |
| ERH_1702 | 843 | 492.9386536 | 1034.521213 | 1.069483 | Up | 5.91E-12 | stage III sporulation protein J; |
| ERH_1560 | 888 | 1363.760188 | 2860.402162 | 1.068628 | Up | 3.78E-11 | hypothetical protein; |
| ERH_0842 | 1293 | 42.40339195 | 88.864273 | 1.067424 | Up | 5.24E-14 | branched-chain amino acid transport system II carrier protein; |
| ERH_1093 | 1272 | 779.8472884 | 1629.618453 | 1.063271 | Up | 2.06E-11 | trigger factor; |
| ERH_1450 | 1806 | 99.14332105 | 206.428464 | 1.058054 | Up | 0 | hypothetical protein; |
| ERH_0503 | 1248 | 175.4250375 | 363.4661542 | 1.050966 | Up | 0 | putative CitXG protein; |
| ERH_0508 | 1155 | 66.65538197 | 137.7147452 | 1.04689 | Up | 1.90E-13 | cysteine desulfurase; |
| ERH_0473 | 435 | 746.9353169 | 1541.252519 | 1.045048 | Up | 3.49E-12 | putative holliday junction resolvase; |
| ERH_0405 | 705 | 133.5093701 | 274.7292174 | 1.041069 | Up | 9.25E-13 | nucleotidyl transferase; |
| ERH_0269 | 726 | 120.1251278 | 247.1199493 | 1.040674 | Up | 1.69E-13 | two-component system response regulator; |
| ERH_1168 | 276 | 3656.381038 | 7498.227784 | 1.036133 | Up | 2.90E-11 | 30S ribosomal protein S19; |
| ERH_1023 | 1077 | 653.8875909 | 1340.460137 | 1.035614 | Up | 0 | cell division protein FtsZ; |
| ERH_0523 | 882 | 241.5065799 | 493.7703479 | 1.031778 | Up | 0 | UDP-N-acetylmuramate dehydrogenase; |
| ERH_0867 | 270 | 43.10601777 | 87.9461304 | 1.028731 | Up | 8.24E-06 | ACT domain-containing protein; |
| ERH_0064 | 855 | 150.4480352 | 306.8304754 | 1.028177 | Up | 1.33E-15 | hypothetical protein; |
| ERH_0109 | 2190 | 494.0349269 | 1007.444075 | 1.028015 | Up | 6.83E-12 | ribonuclease R; |
| ERH_0634 | 249 | 388.622157 | 790.1578512 | 1.023773 | Up | 0 | hypothetical protein; |
| ERH_0329 | 453 | 107.1903916 | 217.7602682 | 1.022565 | Up | 0 | hypothetical protein; |
| ERH_1435 | 1044 | 189.8366185 | 385.5125524 | 1.02202 | Up | 1.05E-12 | ribosomal RNA large subunit methyltransferase RlmN; |
| ERH_1536 | 273 | 141.4171657 | 287.0097647 | 1.021143 | Up | 0 | hypothetical protein; |
| ERH_1497 | 846 | 655.3888235 | 1328.43411 | 1.019304 | Up | 0 | spermidine/putrescine ABC transporter permease; |
| ERH_0650 | 1797 | 143.8455376 | 291.5567358 | 1.019256 | Up | 2.11E-12 | putative endo-beta-N-acetylglucosaminidase; |
| ERH_0772 | 912 | 119.5546877 | 241.6193187 | 1.015065 | Up | 1.38E-12 | prolyl aminopeptidase; |
| ERH_0746 | 813 | 136.9266872 | 276.6724682 | 1.014775 | Up | 4.75E-13 | triphosphoribosyl-dephospho-CoA synthase; |
| ERH_1308 | 336 | 45.43501568 | 91.54000257 | 1.010598 | Up | 3.58E-06 | ArsR family transcriptional regulator; |
| ERH_1498 | 1044 | 1990.697457 | 4008.811594 | 1.009901 | Up | 0 | spermidine/putrescine ABC transporter ATP-binding protein; |
| ERH_0752 | 894 | 111.4952474 | 224.492396 | 1.009684 | Up | 1.15E-12 | citrate lyase subunit beta; |
| ERH_0924 | 1203 | 1105.529473 | 2224.947108 | 1.009034 | Up | 3.59E-11 | acetate kinase; |
| ERH_0885 | 1779 | 72.19624835 | 144.8052408 | 1.004118 | Up | 0 | 1-deoxy-D-xylulose-5-phosphate synthase; |
| ERH_1169 | 837 | 1356.870698 | 2714.160137 | 1.000223 | Up | 2.40E-11 | 50S ribosomal protein L2; |
| ERH_1284 | 240 | 10.54003544 | #VALUE! | -13.3636 | Down | 0.0001415 | hypothetical protein; |
| ERH_0605 | 174 | 300.900646 | 5.949466361 | -5.66038 | Down | 1.88E-64 | hypothetical protein; |
| ERH_0463 | 393 | 3239.577119 | 156.9996144 | -4.36697 | Down | 0 | heat shock protein Hsp20; |
| ERH_0808 | 1539 | 386.3539225 | 21.18883414 | -4.18855 | Down | 0 | hypothetical protein; |
| ERH_0094 | 1881 | 1078.578193 | 67.92643043 | -3.98901 | Down | 0 | surface protective antigen SpaA.1; |
| ERH_1265 | 318 | 269.7135231 | 19.68527583 | -3.77624 | Down | 1.17E-83 | hypothetical protein; |
| ERH_0691 | 189 | 1120.176332 | 83.18757761 | -3.75121 | Down | 2.05E-203 | hypothetical protein; |
| ERH_0203 | 1236 | 484.6257016 | 39.16905856 | -3.62908 | Down | 0 | Na+ efflux pump ABC transporter permease; |
| ERH_0807 | 693 | 338.2744326 | 27.98599272 | -3.59542 | Down | 3.68E-218 | ABC transporter ATP-binding protein; |
| ERH_0692 | 270 | 110.4772063 | 9.55531454 | -3.5313 | Down | 1.79E-28 | hypothetical protein; |
| ERH_1478 | 1098 | 363.2954495 | 35.7022871 | -3.34705 | Down | 0 | ABC transporter permease; |
| ERH_1247 | 822 | 330.5614603 | 32.97059006 | -3.32567 | Down | 4.40E-230 | RpiR family phosphosugar-binding transcriptional regulator; |
| ERH_0481 | 264 | 1982.210186 | 209.6674074 | -3.24094 | Down | 0 | 30S ribosomal protein S20; |
| ERH_1518 | 663 | 178.8980525 | 21.28666796 | -3.07112 | Down | 2.71E-96 | amino acid ABC transporter permease; |
| ERH_1519 | 735 | 54.99781687 | 6.558187996 | -3.06801 | Down | 1.17E-34 | polar amino acid ABC transporter ATP-binding protein; |
| ERH_1517 | 822 | 139.9917466 | 17.11010237 | -3.03242 | Down | 2.79E-92 | amino acid ABC transporter amino acid-binding protein; |
| ERH_1312 | 726 | 1187.129752 | 145.9911234 | -3.02353 | Down | 0 | dipeptidase E; |
| ERH_1477 | 969 | 185.6753788 | 23.12803003 | -3.00507 | Down | 3.73E-145 | ABC transporter permease; |
| ERH_0202 | 909 | 384.4721929 | 49.61179251 | -2.95412 | Down | 1.26E-269 | ABC transporter ATP-binding protein; |
| ERH_1479 | 1554 | 398.6965908 | 52.71569631 | -2.91899 | Down | 0 | ABC transporter ATP-binding protein; |
| ERH_1270 | 402 | 91.96587636 | 12.47357438 | -2.88222 | Down | 2.28E-28 | XRE family transcriptional regulator; |
| ERH_0628 | 231 | 24.18709891 | 3.501154361 | -2.78834 | Down | 1.11E-05 | hypothetical protein; |
| ERH_0848 | 231 | 166.8624806 | 24.15791395 | -2.78809 | Down | 8.61E-28 | hypothetical protein; |
| ERH_1689 | 399 | 868.2723812 | 142.5581414 | -2.6066 | Down | 4.33E-226 | Fur family ferric uptake regulator; |
| ERH_0316 | 1308 | 392.342363 | 64.50366966 | -2.60466 | Down | 0 | branched-chain amino acid transport system II carrier protein; |
| ERH_0693 | 996 | 25.87840048 | 4.466140237 | -2.53465 | Down | 1.59E-18 | hypothetical protein; |
| ERH_1362 | 1344 | 354.2377232 | 64.10566663 | -2.46619 | Down | 1.33E-295 | protein PASTA domain-containing protein; |
| ERH_1203 | 396 | 20747.73988 | 3832.665647 | -2.43653 | Down | 0 | hypothetical protein; |
| ERH_1381 | 846 | 70.65212484 | 13.15440246 | -2.42519 | Down | 7.57E-40 | ABC transporter ATP-binding protein; |
| ERH_0809 | 159 | 123.2186766 | 23.39835494 | -2.39674 | Down | 1.09E-14 | hypothetical protein; |
| ERH_0382 | 642 | 1974.105916 | 375.2069183 | -2.39544 | Down | 0 | pyroglutamyl-peptidase I; |
| ERH_1349 | 408 | 1233.69309 | 242.1545011 | -2.34898 | Down | 9.45E-282 | organic hydroperoxide resistance protein; |
| ERH_1415 | 501 | 1875.268426 | 373.8421407 | -2.3266 | Down | 0 | N-acetyltransferase GCN5; |
| ERH_0825 | 174 | 133.6709321 | 26.86584543 | -2.31484 | Down | 1.11E-14 | hypothetical protein; |
| ERH_0268 | 111 | 561.6272946 | 113.0087039 | -2.31318 | Down | 4.01E-36 | hypothetical protein; |
| ERH_0811 | 330 | 574.7433002 | 115.8990135 | -2.31005 | Down | 3.45E-109 | hypothetical protein; |
| ERH_1277 | 171 | 107.8771149 | 22.08759116 | -2.28808 | Down | 2.08E-11 | hypothetical protein; |
| ERH_0389 | 381 | 641.5468943 | 131.8444631 | -2.28272 | Down | 5.35E-134 | hypothetical protein; |
| ERH_0601 | 420 | 36.03811689 | 7.413722483 | -2.28125 | Down | 6.67E-11 | hypothetical protein; |
| ERH_0629 | 600 | 1364.605023 | 282.9068681 | -2.27008 | Down | 0 | hypothetical protein; |
| ERH_0487 | 294 | 32.63994766 | 6.877267494 | -2.24673 | Down | 9.30E-07 | hypothetical protein; |
| ERH_1246 | 1296 | 1111.189447 | 242.1497425 | -2.19813 | Down | 0 | sugar ABC transporter sugar-binding protein; |
| ERH_1242 | 294 | 336.592622 | 77.10827671 | -2.12605 | Down | 3.53E-53 | hypothetical protein; |
| ERH_0768 | 1821 | 386.1834726 | 89.27094641 | -2.11302 | Down | 0 | choline-binding protein; |
| ERH_1442 | 1038 | 155.114726 | 36.44925463 | -2.08937 | Down | 6.79E-82 | glycosyl transferase family protein; |
| ERH_0462 | 1320 | 11296.87123 | 2654.588098 | -2.08936 | Down | 0 | product aminopeptidase C; |
| ERH_1396 | 414 | 160.8905313 | 38.01612829 | -2.0814 | Down | 4.62E-36 | PTS system fructose subfamily transporter subunit IIA; |
| ERH_0162 | 489 | 2888.992747 | 690.1823894 | -2.06552 | Down | 0 | thiol peroxidase; |
| ERH_0250 | 543 | 33.56512484 | 8.057956061 | -2.05848 | Down | 1.12E-10 | hypothetical protein; |
| ERH_0611 | 225 | 266.1423037 | 64.01851057 | -2.05564 | Down | 1.64E-30 | hypothetical protein; |
| ERH_1066 | 438 | 1017.344037 | 246.1766834 | -2.04704 | Down | 1.03E-223 | N-acetyltransferase GCN5; |
| ERH_0798 | 1401 | 1331.044667 | 332.1848631 | -2.0025 | Down | 0 | arginine/ornithine antiporter; |
| ERH_0381 | 417 | 330.0777249 | 82.46688192 | -2.00092 | Down | 1.76E-68 | BtrG family protein; |
| ERH_1480 | 1092 | 401.3178173 | 100.5331208 | -1.99707 | Down | 7.50E-210 | basic membrane lipoprotein; |
| ERH_1395 | 465 | 75.26244236 | 19.04520113 | -1.9825 | Down | 9.12E-19 | PTS system sorbose subfamily transporter subunit IIB; |
| ERH_0140 | 387 | 110.9994354 | 28.44269068 | -1.96442 | Down | 2.58E-21 | XRE family transcriptional regulator; |
| ERH_0222 | 1566 | 118.6403751 | 30.60531342 | -1.95474 | Down | 2.90E-91 | PTS system glucose-specific transporter subunit IIBC s; |
| ERH_1481 | 903 | 386.7030816 | 101.136505 | -1.93492 | Down | 1.88E-170 | ribokinase; |
| ERH_1278 | 213 | 41.93939872 | 11.1254431 | -1.91444 | Down | 2.95E-05 | hypothetical protein; |
| ERH_0941 | 318 | 2384.064243 | 642.0550963 | -1.89265 | Down | 0 | hypothetical protein; |
| ERH_0588 | 381 | 1093.191915 | 298.3745026 | -1.87335 | Down | 3.35E-182 | hypothetical protein; |
| ERH_0208 | 231 | 1598.522165 | 438.2756688 | -1.86683 | Down | 6.32E-155 | D-alanine--poly(phosphoribitol) ligase subunit 2; |
| ERH_1248 | 876 | 218.7060917 | 60.42684871 | -1.85573 | Down | 2.11E-85 | ROK family protein; |
| ERH_1081 | 984 | 198.4120243 | 54.85494778 | -1.85481 | Down | 6.78E-88 | aldose 1-epimerase; |
| ERH_1662 | 807 | 8314.662897 | 2306.901432 | -1.8497 | Down | 0 | hypothetical protein; |
| ERH_0041 | 474 | 1763.920047 | 489.9010232 | -1.84822 | Down | 0 | hypothetical protein; |
| ERH_1002 | 954 | 620.4769245 | 172.8676832 | -1.84371 | Down | 9.61E-255 | ABC transporter permease; |
| ERH_0786 | 264 | 750.0015352 | 209.8511107 | -1.83753 | Down | 2.84E-82 | hypothetical protein; |
| ERH_1404 | 1056 | 87.05963106 | 24.37801334 | -1.83642 | Down | 4.07E-42 | butyrate kinase; |
| ERH_1243 | 699 | 198.2231604 | 56.04667549 | -1.82242 | Down | 2.04E-62 | N-acylglucosamine-6-phosphate 2-epimerase; |
| ERH_0899 | 336 | 539.841394 | 155.6153351 | -1.79455 | Down | 5.20E-74 | ribosome-binding factor A; |
| ERH_0823 | 696 | 75.51177144 | 22.17139236 | -1.768 | Down | 3.98E-23 | peptidase A24A domain-containing protein; |
| ERH_1294 | 210 | 61.03009483 | 18.02410015 | -1.75959 | Down | 1.73E-06 | hypothetical protein; |
| ERH_1475 | 633 | 70.68283854 | 20.87728717 | -1.75943 | Down | 9.41E-21 | hypothetical protein; |
| ERH_0292 | 345 | 640.4978946 | 189.6264575 | -1.75603 | Down | 2.80E-88 | hypothetical protein; |
| ERH_0246 | 813 | 14.37274477 | 4.257768979 | -1.75517 | Down | 1.05E-06 | transposase of ISErh3; |
| ERH_1476 | 948 | 87.32646173 | 26.1485278 | -1.73969 | Down | 3.16E-37 | inosine/uridine-preferring nucleoside hydrolase; |
| ERH_1083 | 1011 | 417.3316083 | 126.1629927 | -1.72591 | Down | 1.95E-163 | beta-methylgalactoside transporter inner membrane protein; |
| ERH_1244 | 834 | 127.7407924 | 38.91597328 | -1.71478 | Down | 1.06E-43 | sugar ABC transporter permease; |
| ERH_1474 | 1014 | 91.77748081 | 28.03563727 | -1.71088 | Down | 3.95E-40 | inosine/uridine-preferring nucleoside hydrolase; |
| ERH_1397 | 813 | 55.49847196 | 17.0706991 | -1.70093 | Down | 3.22E-20 | sugar phosphate isomerase/epimerase; |
| ERH_0383 | 954 | 1098.418238 | 338.8684053 | -1.69663 | Down | 0 | hypothetical protein; |
| ERH_0515 | 729 | 27.51913316 | 8.49821004 | -1.6952 | Down | 1.40E-09 | DNA protecting protein DprA; |
| ERH_0228 | 1446 | 331.0961862 | 102.3156068 | -1.69422 | Down | 2.25E-181 | ABC transporter substrate-binding protein; |
| ERH_1573 | 966 | 667.1160967 | 206.6627479 | -1.69066 | Down | 1.89E-241 | NAD-dependent epimerase/dehydratase; |
| ERH_0279 | 639 | 16.1564499 | 5.012104636 | -1.68862 | Down | 3.78E-06 | transposase of ISErh3; |
| ERH_0267 | 153 | 1607.397307 | 499.5365934 | -1.68606 | Down | 1.48E-95 | hypothetical protein; |
| ERH_1050 | 321 | 2688.990419 | 837.5665561 | -1.68279 | Down | 0 | hypothetical protein; |
| ERH_1231 | 1176 | 818.5467041 | 255.8070587 | -1.67801 | Down | 0 | class I and II aminotransferase; |
| ERH_1313 | 183 | 72.07814316 | 22.58363228 | -1.67428 | Down | 3.53E-06 | hypothetical protein; |
| ERH_1592 | 291 | 2280.992783 | 716.3045404 | -1.67102 | Down | 2.80E-245 | 30S ribosomal protein S6; |
| ERH_0180 | 135 | 94.19479063 | 29.59507879 | -1.67029 | Down | 5.73E-06 | hypothetical protein; |
| ERH_1571 | 354 | 319.5305314 | 100.8676673 | -1.66349 | Down | 2.19E-41 | ArsC family protein; |
| ERH_1439 | 393 | 283.3564938 | 89.47928979 | -1.66299 | Down | 1.46E-41 | glycerol-3-phosphate cytidylyltransferase; |
| ERH_1617 | 387 | 126.2495774 | 39.99960154 | -1.65822 | Down | 7.30E-20 | hypothetical protein; |
| ERH_1678 | 2457 | 64.19181194 | 20.38879583 | -1.65461 | Down | 1.30E-62 | hypothetical protein; |
| ERH_0061 | 681 | 664.1438454 | 211.3248217 | -1.65203 | Down | 5.42E-158 | RelA/SpoT domain-containing protein; |
| ERH_0714 | 636 | 32.7812566 | 10.4783679 | -1.64546 | Down | 3.51E-09 | ABC transporter ATP-binding protein; |
| ERH_0998 | 291 | 189.8958222 | 61.06033875 | -1.6369 | Down | 8.09E-21 | single-strand DNA-binding protein; |
| ERH_0958 | 372 | 20.89655578 | 6.761482236 | -1.62785 | Down | 0.0001394 | hypothetical protein; |
| ERH_0179 | 399 | 1438.421584 | 466.8344898 | -1.6235 | Down | 4.18E-208 | putative restriction endonuclease; |
| ERH_0983 | 984 | 501.2053513 | 162.8383248 | -1.62196 | Down | 9.37E-176 | phosphate starvation-inducible protein PhoH; |
| ERH_0182 | 321 | 23.01255086 | 7.533386387 | -1.61105 | Down | 0.0003796 | hypothetical protein; |
| ERH_0824 | 174 | 65.55839311 | 21.52061645 | -1.60706 | Down | 9.97E-06 | hypothetical protein; |
| ERH_1580 | 267 | 5196.331183 | 1707.624621 | -1.6055 | Down | 0 | phosphocarrier protein HPr; |
| ERH_1082 | 1164 | 352.0623945 | 116.305567 | -1.59791 | Down | 1.33E-146 | galactokinase; |
| ERH_1685 | 603 | 1724.062526 | 571.0202443 | -1.5942 | Down | 0 | phosphoglycerate mutase family protein; |
| ERH_1454 | 3042 | 169.2497406 | 56.55265878 | -1.58149 | Down | 1.80E-178 | LPXTG-motif cell wall anchor domain-containing protein; |
| ERH_0774 | 249 | 5516.753232 | 1846.596825 | -1.57895 | Down | 0 | hypothetical protein; |
| ERH_0799 | 1293 | 831.7645993 | 280.4790826 | -1.56828 | Down | 0 | AraC family transcriptional regulator; |
| ERH_1422 | 300 | 23.50594991 | 7.952950877 | -1.56346 | Down | 0.0006194 | hypothetical protein; |
| ERH_1618 | 1278 | 171.3290367 | 57.98068985 | -1.56313 | Down | 2.53E-78 | amino acid permease; |
| ERH_0187 | 318 | 31.64295508 | 10.75815223 | -1.55645 | Down | 3.48E-05 | hypothetical protein; |
| ERH_0877 | 1005 | 26.55720881 | 9.045315376 | -1.55386 | Down | 3.23E-11 | putative spermidine/putrescine ABC transporter ATP-binding protein; |
| ERH_0118 | 657 | 880.1869065 | 302.3090641 | -1.54179 | Down | 5.56E-190 | OmpR family two component system response regulator CssR; |
| ERH_1361 | 693 | 176.860928 | 60.83864454 | -1.53956 | Down | 1.73E-42 | ABC transporter ATP-binding protein; |
| ERH_1174 | 531 | 79.2284147 | 27.65959515 | -1.51824 | Down | 2.24E-15 | hypothetical protein; |
| ERH_1500 | 309 | 14573.36462 | 5098.523325 | -1.51518 | Down | 0 | thioredoxin; |
| ERH_1146 | 363 | 12370.77387 | 4334.092695 | -1.51313 | Down | 0 | 50S ribosomal protein L17; |
| ERH_0710 | 636 | 14.75367541 | 5.188347836 | -1.50773 | Down | 7.47E-05 | ABC transporter ATP-binding protein; |
| ERH_0597 | 261 | 87.04159944 | 30.89432088 | -1.49436 | Down | 1.26E-08 | hypothetical protein; |
| ERH_1408 | 1245 | 23.61346515 | 8.412491435 | -1.489 | Down | 2.97E-12 | pyruvate dehydrogenase complex, E2 component, dihydrolipoamide acetyltransferase; |
| ERH_1315 | 1032 | 435.0409052 | 155.1231243 | -1.48774 | Down | 8.00E-146 | peptidase, M42 family; |
| ERH_0184 | 837 | 32.64216583 | 11.64357665 | -1.4872 | Down | 3.56E-11 | putative type II/IV secretion system protein; |
| ERH_0062 | 291 | 777.4811517 | 278.0659744 | -1.48338 | Down | 7.43E-70 | HesB/YadR/YfhF-like protein; |
| ERH_0599 | 405 | 51.25405859 | 18.51189266 | -1.46921 | Down | 4.63E-08 | RusA-like endodeoxyribonuclease; |
| ERH_0224 | 1227 | 45.71136235 | 16.51152421 | -1.46908 | Down | 2.19E-20 | beta-N-acetylhexosaminidase; |
| ERH_0324 | 417 | 32.31617214 | 11.67583906 | -1.46873 | Down | 2.98E-06 | hypothetical protein; |
| ERH_1332 | 576 | 152.6487945 | 55.26573389 | -1.46576 | Down | 1.25E-28 | xanthine phosphoribosyltransferase; |
| ERH_0384 | 684 | 1117.187784 | 404.6793623 | -1.46502 | Down | 2.70E-246 | hypothetical protein; |
| ERH_1530 | 612 | 103.8431322 | 37.76890903 | -1.45913 | Down | 6.89E-22 | RNA polymerase factor sigma-70; |
| ERH_0154 | 1605 | 703.530712 | 256.2760008 | -1.45692 | Down | 0 | hypothetical protein; |
| ERH_1296 | 1533 | 4401.577055 | 1606.716372 | -1.45391 | Down | 0 | GMP synthase large subunit; |
| ERH_0299 | 3597 | 51.01855964 | 18.67117058 | -1.45021 | Down | 3.39E-61 | neuraminidase; |
| ERH_1282 | 294 | 170.6858381 | 62.5006552 | -1.4494 | Down | 8.27E-17 | hypothetical protein; |
| ERH_0124 | 756 | 122.3277753 | 44.93155909 | -1.44495 | Down | 2.08E-29 | type III pantothenate kinase; |
| ERH_0767 | 357 | 182.3331123 | 67.10288463 | -1.44213 | Down | 2.98E-20 | hypothetical protein; |
| ERH_1380 | 1863 | 46.94403061 | 17.29106618 | -1.44091 | Down | 3.95E-30 | hypothetical protein; |
| ERH_0016 | 879 | 354.2576523 | 131.1508982 | -1.43357 | Down | 1.99E-92 | hypothetical protein; |
| ERH_1409 | 981 | 33.125805 | 12.26752854 | -1.43311 | Down | 6.70E-13 | pyruvate dehydrogenase complex, E1 component subunit beta; |
| ERH_0418 | 552 | 1033.942255 | 383.3299814 | -1.4315 | Down | 9.26E-161 | hypothetical protein; |
| ERH_1097 | 471 | 293.7267418 | 109.7416552 | -1.42036 | Down | 9.87E-41 | phosphodiesterase; |
| ERH_0410 | 957 | 455.1846998 | 170.466273 | -1.41697 | Down | 1.05E-130 | ABC transporter substrate-binding protein; |
| ERH_1067 | 708 | 1030.537781 | 387.9335204 | -1.40952 | Down | 3.80E-216 | SpoU rRNA methylase family protein; |
| ERH_0396 | 522 | 5509.467443 | 2075.541187 | -1.40843 | Down | 0 | PfpI family intracellular protease; |
| ERH_1263 | 678 | 44.53404136 | 16.78373578 | -1.40784 | Down | 3.52E-11 | ABC transporter ATP-binding protein; |
| ERH_1330 | 603 | 1678.512658 | 633.0380309 | -1.40682 | Down | 3.56E-284 | hypothetical protein; |
| ERH_1382 | 1038 | 65.33333687 | 24.79302332 | -1.39789 | Down | 4.90E-22 | hypothetical protein; |
| ERH_1690 | 975 | 663.3104295 | 252.0780435 | -1.39581 | Down | 9.09E-185 | guanosine monophosphate reductase 2; |
| ERH_0626 | 372 | 94.36370252 | 35.91648885 | -1.39359 | Down | 5.57E-12 | phage holin; |
| ERH_1353 | 1005 | 665.6899745 | 253.3817814 | -1.39354 | Down | 2.36E-191 | aspartate--ammonia ligase; |
| ERH_1239 | 609 | 20.55076374 | 7.861964549 | -1.38623 | Down | 4.45E-05 | VanZ family protein; |
| ERH_0408 | 1746 | 2603.603842 | 999.3299563 | -1.38148 | Down | 0 | oligoendopeptidase F; |
| ERH_1202 | 1803 | 875.2502153 | 336.501217 | -1.37908 | Down | 0 | oligoendopeptidase F; |
| ERH_0004 | 927 | 168.9691766 | 65.11151175 | -1.37578 | Down | 4.13E-45 | 1-phosphofructokinase; |
| ERH_0149 | 849 | 164.4768761 | 63.39657565 | -1.37541 | Down | 3.33E-42 | AraC family transcriptional regulator; |
| ERH_0081 | 987 | 115.651835 | 44.6176336 | -1.3741 | Down | 1.11E-34 | bleomycin resistance protein; |
| ERH_0284 | 477 | 22.76668596 | 8.867629072 | -1.3603 | Down | 6.97E-05 | PTS system transporter subunit IIB; |
| ERH_1345 | 357 | 2008.185647 | 784.1669631 | -1.35666 | Down | 3.12E-191 | alkylhydroperoxidase-like protein; |
| ERH_0602 | 183 | 691.1978904 | 271.0919255 | -1.35032 | Down | 1.11E-34 | hypothetical protein; |
| ERH_0627 | 1053 | 71.71207042 | 28.23408722 | -1.34478 | Down | 2.08E-23 | CHAP domain-containing protein; |
| ERH_1075 | 2589 | 711.8304637 | 280.5129975 | -1.34347 | Down | 0 | magnesium-translocating P-type ATPase; |
| ERH_1515 | 693 | 783.7919072 | 310.459355 | -1.33607 | Down | 8.76E-144 | hypothetical protein; |
| ERH_1698 | 273 | 280.0009529 | 111.2134015 | -1.3321 | Down | 1.73E-20 | S4 RNA-binding domain-containing protein; |
| ERH_1403 | 897 | 59.88089809 | 23.85735085 | -1.32766 | Down | 1.81E-16 | LysR family transcriptional regulator; |
| ERH_0352 | 1077 | 26.83090497 | 10.69344654 | -1.32717 | Down | 2.30E-10 | sugar isomerase; |
| ERH_1098 | 759 | 125.3404415 | 50.43347327 | -1.3134 | Down | 7.65E-26 | glutamate racemase; |
| ERH_0950 | 285 | 22507.88761 | 9076.474769 | -1.31023 | Down | 0 | DNA-binding protein HU; |
| ERH_0722 | 444 | 23.17838248 | 9.3628662 | -1.30776 | Down | 0.0003964 | PTS system ascorbate-specific transporter subunit IIA; |
| ERH_1406 | 1050 | 30.22386709 | 12.22398884 | -1.30597 | Down | 4.69E-11 | putative regulatory protein; |
| ERH_1377 | 1722 | 29.32596581 | 11.9530744 | -1.2948 | Down | 1.09E-16 | ABC transporter permease/ATP-binding protein; |
| ERH_1410 | 1098 | 26.53445346 | 10.82046412 | -1.2941 | Down | 4.59E-10 | pyruvate dehydrogenase complex, E1 component subunit alpha; |
| ERH_0086 | 942 | 2632.40893 | 1074.32259 | -1.29296 | Down | 0 | L-lactate dehydrogenase; |
| ERH_0235 | 1296 | 25.30596334 | 10.33430989 | -1.29204 | Down | 1.98E-11 | hypothetical protein; |
| ERH_0237 | 1392 | 31.9488848 | 13.06697264 | -1.28984 | Down | 3.34E-15 | beta-glucosidase; |
| ERH_1266 | 291 | 242.9707417 | 99.41434322 | -1.28926 | Down | 1.71E-19 | co-chaperonin GroES; |
| ERH_1115 | 519 | 64.89768653 | 26.55380347 | -1.28925 | Down | 1.18E-10 | putative tryptophan transport protein; |
| ERH_0883 | 1179 | 404.3757306 | 165.4851996 | -1.28899 | Down | 1.16E-121 | DNA polymerase IV; |
| ERH_0165 | 4560 | 39.2158282 | 16.13108871 | -1.28159 | Down | 5.25E-51 | pectin lyase fold-containing protein; |
| ERH_0876 | 1029 | 54.92035132 | 22.65974938 | -1.27721 | Down | 5.28E-17 | putative spermidine/putrescine ABC transporter substrate-binding protein; |
| ERH_0128 | 489 | 375.2380794 | 155.567856 | -1.27026 | Down | 2.09E-46 | N-acetyltransferase GCN5; |
| ERH_0227 | 912 | 137.140511 | 56.89749041 | -1.26922 | Down | 1.34E-32 | sugar ABC transporter permease; |
| ERH_0249 | 354 | 38.98139982 | 16.26689272 | -1.26085 | Down | 8.15E-05 | hypothetical protein; |
| ERH_0787 | 765 | 324.7627825 | 135.5244962 | -1.26083 | Down | 3.37E-62 | biotin/lipoate A/B protein ligase family protein; |
| ERH_1393 | 825 | 57.02292088 | 23.87095634 | -1.25629 | Down | 6.89E-14 | PTS system mannose/fructose/sorbose family transporter subunit IID; |
| ERH_0005 | 1857 | 386.5048196 | 162.1288331 | -1.25335 | Down | 6.92E-172 | PTS system fructose-specific transporter subunit IIABC; |
| ERH_0123 | 615 | 79.45492795 | 33.42898727 | -1.24904 | Down | 9.53E-14 | hypothetical protein; |
| ERH_1591 | 474 | 11101.46272 | 4675.124022 | -1.24767 | Down | 0 | single-strand DNA-binding protein; |
| ERH_1472 | 1650 | 155.7530635 | 65.80437518 | -1.24301 | Down | 1.07E-62 | internalin-like protein; |
| ERH_0561 | 4695 | 24.82992872 | 10.49249326 | -1.24272 | Down | 5.52E-34 | glycoside hydrolase; |
| ERH_0794 | 483 | 416.4837262 | 177.2256681 | -1.23267 | Down | 2.17E-47 | methylated DNA-protein cysteine methyltransferase; |
| ERH_1405 | 897 | 26.31089776 | 11.19828386 | -1.23238 | Down | 4.39E-08 | phosphate butyryltransferase; |
| ERH_1431 | 726 | 25.8124423 | 10.99521938 | -1.23119 | Down | 1.24E-06 | hypothetical protein; |
| ERH_0150 | 3105 | 30.44273734 | 12.97419488 | -1.23045 | Down | 4.52E-28 | hyaluronidase; |
| ERH_1697 | 321 | 593.4155436 | 253.3638099 | -1.22783 | Down | 2.40E-42 | septum formation initiator; |
| ERH_0356 | 255 | 1253.104866 | 535.0242003 | -1.22783 | Down | 1.70E-71 | glutaredoxin-like protein NrdH; |
| ERH_0880 | 912 | 224.968497 | 96.14762819 | -1.2264 | Down | 2.18E-50 | hypothetical protein; |
| ERH_0057 | 774 | 1127.519116 | 482.1244616 | -1.22567 | Down | 2.34E-208 | pyrroline-5-carboxylate reductase; |
| ERH_0091 | 339 | 379.9867682 | 162.5409679 | -1.22515 | Down | 4.44E-31 | hypothetical protein; |
| ERH_1590 | 234 | 3014.317847 | 1292.610612 | -1.22154 | Down | 1.98E-157 | 30S ribosomal protein S18; |
| ERH_1297 | 1143 | 959.4883322 | 411.5370444 | -1.22124 | Down | 7.90E-269 | inosine-5'-monophosphate dehydrogenase; |
| ERH_1581 | 456 | 594.5441849 | 255.9677391 | -1.21582 | Down | 6.24E-62 | SPOUT methyltransferase superfamily protein; |
| ERH_1359 | 447 | 3961.349266 | 1706.954951 | -1.21457 | Down | 0 | hypothetical protein; |
| ERH_1641 | 357 | 43.26207527 | 18.64486863 | -1.21432 | Down | 2.48E-05 | hypothetical protein; |
| ERH_1147 | 960 | 4802.579164 | 2071.854533 | -1.21289 | Down | 0 | DNA-directed RNA polymerase subunit alpha; |
| ERH_1245 | 897 | 59.62634228 | 25.77776507 | -1.20982 | Down | 7.35E-15 | sugar ABC transporter permease; |
| ERH_0781 | 456 | 66.06388334 | 28.57314925 | -1.2092 | Down | 1.48E-08 | N-acetyltransferase GCN5; |
| ERH_0236 | 2679 | 36.56970737 | 15.8312318 | -1.20788 | Down | 3.03E-27 | alpha-mannosidase; |
| ERH_0797 | 936 | 2323.986375 | 1006.536063 | -1.2072 | Down | 0 | carbamate kinase; |
| ERH_1043 | 1446 | 1366.084357 | 595.5690373 | -1.19771 | Down | 0 | V-type ATPase subunit B; |
| ERH_0360 | 507 | 19760.73783 | 8634.469958 | -1.19446 | Down | 0 | hypothetical protein; |
| ERH_0053 | 396 | 138.0218462 | 60.31011655 | -1.19442 | Down | 6.65E-14 | hypothetical protein; |
| ERH_0460 | 1812 | 2032.654876 | 891.2241162 | -1.18951 | Down | 0 | glucosamine--fructose-6-phosphate aminotransferase; |
| ERH_0447 | 189 | 2927.880889 | 1287.051524 | -1.18579 | Down | 3.55E-121 | hypothetical protein; |
| ERH_1126 | 483 | 530.1118319 | 233.0858394 | -1.18544 | Down | 1.93E-56 | nucleoside 2-deoxyribosyltransferase; |
| ERH_1513 | 1425 | 184.7010769 | 81.62598163 | -1.17809 | Down | 4.19E-63 | aromatic-L-amino-acid decarboxylase; |
| ERH_0088 | 1152 | 1726.858419 | 764.339745 | -1.17586 | Down | 0 | tRNA-guanine transglycosylase; |
| ERH_1392 | 996 | 116.6366695 | 52.02592755 | -1.16472 | Down | 5.71E-28 | sugar isomerase; |
| ERH_0870 | 318 | 307.8322804 | 138.8637463 | -1.14847 | Down | 2.79E-21 | hypothetical protein; |
| ERH_1407 | 1407 | 27.72588599 | 12.5195748 | -1.14705 | Down | 2.82E-11 | pyruvate dehydrogenase complex, E3 component, dihydrolipoamide dehydrogenase; |
| ERH_0684 | 543 | 32.4458219 | 14.65624218 | -1.14652 | Down | 3.21E-05 | replication initiator protein A N-terminal domain-containing protein; |
| ERH_0084 | 525 | 21.88544343 | 9.920924996 | -1.14143 | Down | 0.0006584 | flavin reductase FMN-binding domain-containing protein; |
| ERH_0982 | 675 | 218.1465337 | 99.04102876 | -1.1392 | Down | 4.42E-33 | two-component system response regulator; |
| ERH_0232 | 1179 | 28.35609805 | 12.98561913 | -1.12674 | Down | 1.32E-09 | Bcr/CflA family drug resistance transporter; |
| ERH_0442 | 1779 | 25748.98824 | 11804.74852 | -1.12515 | Down | 0 | oligopeptide/dipeptide ABC transporter substrate-binding protein; |
| ERH_1491 | 2484 | 55.44072049 | 25.54262722 | -1.11804 | Down | 1.95E-31 | ABC transporter permease; |
| ERH_0879 | 834 | 14.99429532 | 6.962802966 | -1.10667 | Down | 0.0002675 | putative spermidine/putrescine ABC transporter permease; |
| ERH_0694 | 1389 | 30.57738036 | 14.21886354 | -1.10466 | Down | 2.70E-10 | radical SAM domain-containing protein; |
| ERH_1507 | 795 | 378.7117049 | 176.8502556 | -1.09857 | Down | 3.40E-61 | hypothetical protein; |
| ERH_0723 | 1485 | 25.84741917 | 12.07441934 | -1.09807 | Down | 3.23E-10 | PTS system ascorbate-specific transporter subunit IIC; |
| ERH_1233 | 213 | 346.0811944 | 161.8677858 | -1.09629 | Down | 1.52E-15 | hypothetical protein; |
| ERH_0817 | 1089 | 26.33254937 | 12.34316273 | -1.09314 | Down | 3.48E-08 | hypothetical protein; |
| ERH_1227 | 2127 | 729.601438 | 342.0016013 | -1.09311 | Down | 1.00E-307 | hypothetical protein; |
| ERH_0238 | 879 | 48.34355099 | 22.68061829 | -1.09186 | Down | 5.54E-11 | ROK family protein; |
| ERH_1316 | 819 | 325.4910666 | 152.796672 | -1.091 | Down | 2.55E-52 | AraC family transcriptional regulator; |
| ERH_0024 | 852 | 20.67976737 | 9.720462861 | -1.08912 | Down | 1.12E-05 | ABC transporter ATP-binding protein; |
| ERH_1470 | 648 | 26.44028595 | 12.46849326 | -1.08445 | Down | 4.67E-05 | hypothetical protein; |
| ERH_0275 | 1317 | 37.88910542 | 17.90107322 | -1.08174 | Down | 2.01E-12 | uracil permease; |
| ERH_1492 | 681 | 113.6872895 | 53.73972145 | -1.08101 | Down | 1.31E-16 | ABC transporter ATP-binding protein; |
| ERH_0311 | 435 | 71.35081469 | 33.76364589 | -1.07946 | Down | 8.13E-08 | hypothetical protein; |
| ERH_1138 | 183 | 775.7353979 | 367.1265251 | -1.07929 | Down | 7.61E-26 | hypothetical protein; |
| ERH_1210 | 3060 | 23.96797589 | 11.36241877 | -1.07684 | Down | 2.92E-18 | hyaluronidase; |
| ERH_0323 | 825 | 42.84904392 | 20.35171911 | -1.07411 | Down | 7.70E-09 | ABC transporter ATP-binding protein; |
| ERH_0388 | 747 | 39.13610422 | 18.68715901 | -1.06645 | Down | 1.34E-07 | phospholipase D; |
| ERH_1550 | 1491 | 804.9703249 | 384.8295292 | -1.06472 | Down | 1.35E-235 | peptidase, M23B family; |
| ERH_0780 | 6276 | 71.61003393 | 34.25415292 | -1.06388 | Down | 2.09E-88 | beta-galactosidase; |
| ERH_0351 | 489 | 31.88807971 | 15.3154061 | -1.05803 | Down | 0.0001129 | PTS system mannose/fructose/sorbose family transporter subunit IIB; |
| ERH_1398 | 2703 | 31.23187388 | 15.0054803 | -1.05753 | Down | 1.77E-19 | sigma-54 dependent transcription regulator; |
| ERH_1027 | 651 | 291.1083454 | 139.8762041 | -1.05741 | Down | 1.40E-38 | ribulose-phosphate 3-epimerase; |
| ERH_1084 | 1503 | 183.5800659 | 88.42118137 | -1.05395 | Down | 4.73E-52 | galactose/methyl galactoside ABC transporter ATP-binding protein; |
| ERH_0621 | 2601 | 31.87251129 | 15.36072212 | -1.05307 | Down | 1.42E-19 | tape measure protein; |
| ERH_0727 | 732 | 28.31240669 | 13.64527199 | -1.05303 | Down | 7.59E-06 | L-ribulose-5-phosphate 4-epimerase; |
| ERH_0239 | 894 | 40.0413491 | 19.31465108 | -1.05179 | Down | 4.35E-09 | glucokinase; |
| ERH_0038 | 627 | 57.84215684 | 27.91359091 | -1.05115 | Down | 1.35E-08 | orotate phosphoribosyltransferase; |
| ERH_0728 | 1533 | 29.68573477 | 14.38166502 | -1.04554 | Down | 5.71E-11 | leucine-rich repeat protein; |
| ERH_1460 | 894 | 33.9245304 | 16.45580967 | -1.04373 | Down | 1.78E-07 | hypothetical protein; |
| ERH_0283 | 807 | 42.01697249 | 20.44471754 | -1.03924 | Down | 1.95E-08 | PTS system sorbose-specific transporter subunit IIC; |
| ERH_0494 | 876 | 6150.174928 | 2993.305693 | -1.03889 | Down | 0 | putative peptidylprolyl isomerase; |
| ERH_1545 | 849 | 60.83055824 | 29.63580313 | -1.03746 | Down | 1.92E-11 | aminoglycoside 6-adenylyltranserase; |
| ERH_0732 | 2163 | 42.06739828 | 20.5314826 | -1.03486 | Down | 4.56E-19 | hypothetical protein; |
| ERH_0047 | 1122 | 1735.720549 | 848.2345072 | -1.033 | Down | 0 | manganese/zinc/iron ABC transporter permease; |
| ERH_0247 | 1218 | 24.45683588 | 11.96558608 | -1.03135 | Down | 8.74E-08 | hypothetical protein; |
| ERH_1254 | 498 | 377.1400601 | 184.5546905 | -1.03105 | Down | 4.97E-34 | YbaK/prolyl-tRNA synthetase-associated domain-containing protein; |
| ERH_0025 | 1134 | 31.95628514 | 15.70471822 | -1.0249 | Down | 7.66E-09 | putative ABC transporter permease; |
| ERH_0186 | 726 | 26.81868701 | 13.20104099 | -1.02259 | Down | 1.36E-05 | hypothetical protein; |
| ERH_0083 | 594 | 90.34869743 | 44.52305273 | -1.02095 | Down | 1.98E-11 | phospholipase/carboxylesterase family protein; |
| ERH_0806 | 729 | 83.05292883 | 40.94872407 | -1.02021 | Down | 3.61E-12 | accessory gene regulator A; |
| ERH_0183 | 642 | 56.43673374 | 27.86619627 | -1.01812 | Down | 1.16E-08 | hypothetical protein; |
| ERH_0725 | 645 | 40.88385478 | 20.22552385 | -1.01535 | Down | 2.46E-06 | 3-dehydro-L-gulonate-6-phosphate decarboxylase; |
| ERH_0961 | 993 | 17.75071742 | 8.796289361 | -1.01291 | Down | 4.05E-05 | hypothetical protein; |
| ERH_0069 | 678 | 50.34210692 | 25.02653832 | -1.00831 | Down | 8.92E-08 | HAD-superfamily hydrolase; |
| ERH_1364 | 447 | 64.2918559 | 31.97079337 | -1.00788 | Down | 1.76E-06 | hypothetical protein; |
| ERH_0248 | 1125 | 42.27717091 | 21.0495926 | -1.00609 | Down | 4.88E-11 | hypothetical protein; |
| ERH_1260 | 573 | 90.82658236 | 45.2515686 | -1.00515 | Down | 1.32E-10 | alkylmercury lyase; |
| ERH_0425 | 228 | 366.0296734 | 182.5406544 | -1.00374 | Down | 5.48E-14 | hypothetical protein; |
| ERH_1085 | 1083 | 840.8091805 | 420.2909976 | -1.00039 | Down | 1.18E-153 | D-galactose-binding periplasmic protein; |
| ERH_0978 | 1224 | 30.35856957 | 15.1776498 | -1.00016 | Down | 1.32E-08 | phosphate ABC transporter permease; |
| ERH_1399 | 1437 | 3001.623355 | 1500.748492 | -1.00006 | Down | 0 | PTS system N-acetylglucosamine-specific transporter subunit IIBC; |
